# Clinical Applications of Extracellular Vesicles: Promises and Pitfalls

**DOI:** 10.3390/ijms27031509

**Published:** 2026-02-03

**Authors:** Dragan Primorac, Petar Brlek, Luka Bulić, Nenad Hrvatin, Vedrana Škaro, Petar Projić, Martina Glavan, Ijeoma Oleru, Pierre Rocheteau, Carlo Tremolada, Ariana DeMers, Mary A. Ambach, Don Buford, Tamara Knežević, Dimitrios Kouroupis, Cole Conforti, D. Wood Kimbrough, R. Peter Schnorr, Lindsay Williams, Raminta Vaiciuleviciute, Žan Fortuna, Lara Oprešnik, Blaž Curk, Miomir Knežević, Gordana Kalan Živčec, Adelina Hrkać, Dimitrios Tsoukas, Ilona Uzieliene, Jolita Pachaleva, Eiva Bernotiene, Kristiana Barbato, Neep Patel, Isabella Demirdjian Guanche, Evangelos V. Badiavas, Jana Mešić, Ana Medić Flajšman, Romina Milanič, Danijela Klarić, Vasiliki E. Kalodimou, Massimo Allegri, Johannes Brachmann, Wei Seong Toh, Nancy Duarte Delgado, Ali Mobasheri

**Affiliations:** 1 St. Catherine Specialty Hospital, 10000 Zagreb, Croatia; dragan.primorac@svkatarina.hr (D.P.); luka.bulic@svkatarina.hr (L.B.); nenad.hrvatin@svkatarina.hr (N.H.); tamara.knezevic@svkatarina.hr (T.K.); adelina.surjan@gmail.com (A.H.); jana.mesic@svkatarina.hr (J.M.); ana.medicflajsman@svkatarina.hr (A.M.F.); romina.milanic@svkatarina.hr (R.M.); danijela.klaric@svkatarina.hr (D.K.); 2 International Center for Applied Biological Research, 10000 Zagreb, Croatia; vedrana.skaro@icabs.eu (V.Š.); petar.projic@icabs.eu (P.P.); 3 School of Medicine, Josip Juraj Strossmayer University of Osijek, 31000 Osijek, Croatia; 4 Faculty of Dental Medicine and Health, Josip Juraj Strossmayer University of Osijek, 31000 Osijek, Croatia; 5 Eberly College of Science, The Pennsylvania State University, State College, PA 16802, USA; 6 School of Medicine, University of Split, 21000 Split, Croatia; 7 The Henry C. Lee College of Criminal Justice and Forensic Sciences, University of New Haven, New Haven, CT 06516, USA; 8 Sana Kliniken Oberfranken, 96450 Coburg, Germany; johannes.brachmann@regiomed-kliniken.de; 9 School of Medicine, University of Rijeka, 51000 Rijeka, Croatia; 10 School of Medicine, University of Pittsburgh, Pittsburgh, PA 15213, USA; 11 Gandhinagar Campus, National Forensic Sciences University, Gandhinagar 382007, India; 12 Department of Molecular Biology, Faculty of Science, University of Zagreb, 10000 Zagreb, Croatia; 13 Department of Pediatrics, Clinical Hospital Center Rijeka, 51000 Rijeka, Croatia; 14 Department of Neuroscience, Yale School of Medicine, Yale University, 333 Cedar Street, New Haven, CT 06510, USA; martina.glavan@yale.edu (M.G.); ijeoma.oleru@yale.edu (I.O.); 15 Exogems SA, Biopole, 1066 Epalinges, Switzerland; pierre.rocheteau@gmail.com (P.R.); carlo.tremolada@gmail.com (C.T.); 16 Image Regenerative Clinic, 20122 Milano, Italy; 17 Restore Orthopedics and Sports Medicine, Sonora, CA 95370, USA; arianademers@gmail.com; 18 BioEvolve, San Diego Orthobiologics and Sports Center, San Diego, CA 92024, USA; info@bioevolvesports.com; 19 Texas Orthobiologics, Dallas, TX 75204, USA; info@biologicortho.com; 20 Department of Orthopaedics, UHealth Sports Medicine Institute, Miller School of Medicine, University of Miami, Miami, FL 33146, USA; dxk504@med.miami.edu (D.K.); cmc732@med.miami.edu (C.C.); dwk45@med.miami.edu (D.W.K.); ncp87@med.miami.edu (N.P.); 21 Diabetes Research Institute, Cell Transplant Center, Miller School of Medicine, University of Miami, Miami, FL 33146, USA; kbarbato@miami.edu; 22 FIRST Focused Integrated Regenerative Sports Medicine and Treatments, 8002 Zurich, Switzerland; pschnorr@first-zurich.ch; 23 Department of Immunology and Theranostics, Beckman Research Institute, City of Hope, Duarte, CA 91010, USA; 24 State Research Institute Center for Innovative Medicine, LT-08406 Vilniaus, Lithuania; raminta.vaiciuleviciute@imcentras.lt; 25 GaiaCell, Advanced Cell and Gene Therapy Ltd., Prevale 9, 1236 Trzin, Slovenia; zan.fortuna@gaiacell.net (Ž.F.); lara.opresnik@gaiacell.net (L.O.); blaz.curk@gaiacell.net (B.C.); miomir.knezevic@gaiacell.net (M.K.); gkz@gaiacell.net (G.K.Ž.); 26 Orthopaedic Clinic for Advanced Arthroscopic Sports and Regenerative Surgery Mitera Hospital, 15123 Athens, Greece; info@tsoukas-ortho.gr; 27 Department of Regenerative Medicine, State Research Institute Center for Innovative Medicine, LT-08406 Vilnius, Lithuania; ilona.uzieliene@imcentras.lt (I.U.); jolita.pachaleva@imcentras.lt (J.P.); eiva.bernotiene@imcentras.lt (E.B.); ali.mobasheri@oulu.fi (A.M.); 28 Dr. Phillip Frost Department of Dermatology and Cutaneous Surgery, Miller School of Medicine, University of Miami, Miami, FL 91010, USA; iguanche@miami.edu (I.D.G.); ebadiavas@med.miami.edu (E.V.B.); 29 Medical Innovation Center (MEDIC), School of Medicine, European University Cyprus, Diogenis Str., Engomi, Nicosia 2404, Cyprus; v.kalodimou@euc.ac.cy; 30 Centre Lémanique de Neuromodulation et Thérapie de la Douleur, Ensemble Hospitalier de la Côte (EHC), 1110 Morges, Switzerland; massimo.allegri@ehc.vd.ch; 31 Department of Orthopaedic Surgery, Yong Loo Lin School of Medicine, National University of Singapore, Singapore 117597, Singapore; tohws@nus.edu.sg; 32 Faculty of Dentistry, National University of Singapore, Singapore 117597, Singapore; 33 Research Unit of Health Sciences and Technology, Faculty of Medicine, University of Oulu, 90220 Oulu, Finland; nancy.duartedelgado@oulu.fi; 34 Department of Joint Surgery, First Affiliated Hospital of Sun Yat-sen University, Guangzhou 510000, China; 35 Faculté de Médecine, Université de Liège, 4000 Liège, Belgium

**Keywords:** extracellular vesicles, exosomes, biomarkers, targeted therapy, personalized medicine, regenerative medicine, clinical translation

## Abstract

Extracellular vesicles (EVs) are membrane-bound nanoparticles released by almost all cell types into the extracellular space, acting as important mediators of intercellular communication by transferring proteins, lipids, and nucleic acids horizontally. EVs are generally classified into small EVs (<200 nm), medium/large EVs (>200 nm), microvesicles, and apoptotic bodies, with current classification methods focusing on physical properties, molecular composition, and cellular origin, as detailed in the MISEV2023 guidelines. EVs are highly promising for diagnostic and therapeutic applications due to their intrinsic biocompatibility, stability in biological fluids, capacity to carry diverse molecular cargo, and potential for drug delivery and functionalization to enable targeted delivery and tissue repair. This narrative review discusses the emerging roles of EVs across various medical fields, including obstetrics and gynecology, ophthalmology, otorhinolaryngology, urology, oncology, orthopedics, neurology, immunology, wound healing, chronic pain management, dermatology, and cardiology. In each discipline, EVs show potential as biomarkers for diagnosing physiological or pathological conditions and as carriers for targeted drug delivery and regenerative treatments. Exosomes, a major type of small EVs, have especially attracted attention as versatile nanocarriers for precision medicine. However, translation into clinical practice requires addressing key pitfalls, including the standardization of isolation and characterization protocols, dose definition, GMP-compliant large-scale production, and regulatory approval. Ongoing interdisciplinary collaboration across disciplines and thorough clinical testing will be essential to unlock the full biomedical potential of EVs and establish them as transformative tools in personalized healthcare.

## 1. Introduction

Extracellular vesicles (EVs) are membrane-bound particles released by cells into the extracellular space, serving as carriers of proteins, lipids, and nucleic acids [1]. EVs facilitate intercellular communication by mediating the horizontal transfer of functional biomolecules between diverse cell types and tissues [2]. EVs are classified into subtypes such as small EVs, medium/large EVs, microvesicles, and apoptotic bodies. Modern classification strategies, including those recommended in the MISEV2023 guidelines, emphasize descriptive criteria—such as physical properties, molecular composition, and cellular origin or culture conditions—over strict nomenclature [1]. In this framework, “small EVs” are generally defined as <200 nm, while “medium/large EVs” are >200 nm, with use of biogenesis-based terms (e.g., exosomes, microvesicles, or apoptotic bodies) only in cases where the biogenesis pathway is clearly demonstrated [1]. EVs are highly promising for both diagnostics and therapeutics due to their inherent biocompatibility, stability in biological fluids, and ability to carry diverse molecular cargo, including proteins, lipids, and nucleic acids. Their natural composition not only reflects the physiological state of their parent cells, making them valuable minimally invasive biomarkers, but also enables drug loading and functionalization for targeted delivery, modulation of cellular responses, and tissue regeneration, highlighting their potential as tools supporting precision and personalized medical approaches [3]. In recent years, clinical studies investigating EVs in various pathological conditions have increased significantly, underscoring their potential in personalized medicine and regenerative therapies. In this review, the terms “personalized medicine” and “personalized healthcare” refer to the potential of EV-based approaches to support patient stratification and individualized clinical decision-making. Due to their molecular cargo (e.g., proteins, miRNAs, lipids), EVs may reflect disease subtype, activity, and treatment response, enabling more precise diagnostics and longitudinal monitoring. In addition, engineered or cell-derived EVs may facilitate targeted delivery of therapeutics and reduce systemic adverse effects. Nevertheless, the implementation of EVs in personalized clinical workflows will require standardized analytical pipelines, robust validation in large cohorts, and regulatory-grade manufacturing.

## 2. Materials and Methods

### 2.1. Search Strategy

A comprehensive literature search was performed in PubMed to identify peer-reviewed studies on extracellular vesicles (EVs), without restriction on publication date. The search combined MeSH terms and free-text keywords including “extracellular vesicles”, “exosomes”, “small extracellular vesicles”, “microvesicles”, “apoptotic bodies”, “biomarkers”, “targeted therapy”, “drug delivery”, “regenerative medicine”, “personalized medicine”, “clinical translation”, and “MISEV2023 guidelines” along with discipline-specific terms (e.g., oncology, neurology, immunology, ophthalmology, urology, orthopedics, obstetrics and gynecology, otorhinolaryngology, and wound healing) using Boolean operators.

### 2.2. Eligibility and Study Selection

Only English language, peer-reviewed articles were included. Eligible studies comprised clinical, animal, and in vitro investigations addressing EV biology, diagnostic or therapeutic applications, and translational aspects. Editorials, conference abstracts, and non-peer-reviewed publications were excluded. Overlapping reviews were screened, and the most comprehensive and methodologically robust publications were retained, with preference given to those aligned with MISEV2023 recommendations.

## 3. EVs in Obstetrics and Gynecology (OBGYN)

Extracellular vesicles (EVs) are key mediators of intercellular communication within the female reproductive system, playing crucial roles in processes essential for fertility and pregnancy. In the ovaries, EVs in follicular fluid (FF) carry bioactive molecules—such as microRNAs and proteins—participating in intracellular communication between granulosa cells and cumulus cells [4]. They regulate folliculogenesis, oocyte maturation, and sex hormone production [5]. It was shown that FF EVs are very diverse and are involved in follicular growth, oocyte maturation, and embryo development [6,7]. Oviductal EVs, isolated from human fallopian tubal fluid, participate in embryo development [8]. EVs derived from endometrial cells have been shown to influence uterine environment remodeling and embryo implantation by transferring molecules that regulate immune tolerance and tissue receptivity [9]. Animal models show that in early pregnancy uterine lumen is highly enriched with EVs targeting embryo and organism development and intercellular communication, showing the importance of EVs in the maternal–embryo interface and pregnancy [10]. The endometrial epithelial cell secretome can be modulated by trophoblast cell-derived EVs to improve endometrial receptivity and embryo attachment [11]. Fertile female endometrium cell EVs contain proteins, related to endometrial receptivity, embryo implantation, and early embryo development, and these EVs can be taken up by human blastocysts in vitro [12,13]. The transcriptome of uterine fluid EVs highly correlates with endometrial tissue and contains genes regulating cell adhesion and implantation [14]. Small EVs, released by endometrial epithelial cells during early pregnancy affect embryo implantation by regulating trophoblast migration and proliferation [15]. Placental EVs can be found in maternal blood during the first trimester with placental-type alkaline phosphatase (PLAP) serving as a marker of placental EVs and an important mediator of maternal–fetal communication [16,17].

### 3.1. Diagnostic Value

**Figure 1:**
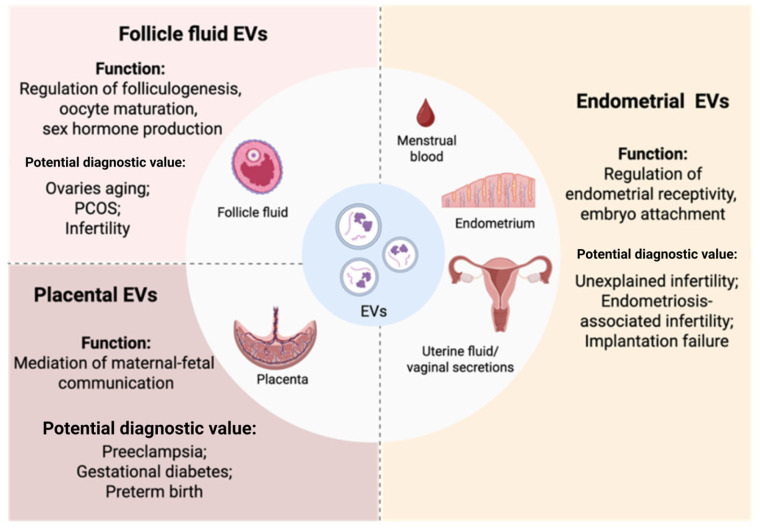
Physiological function and diagnostic value of EVs detected in fluids and tissues of the female reproductive tract (original figure created with Biorender.com). PCOS: polycystic ovary syndrome.

For clarity, this section is structured to highlight EV biomarker evidence, while therapeutic findings are summarized separately. Emerging evidence suggests that EVs, isolated from cells or biofluids of the female reproductive tract, hold significant promise as biomarkers of reproductive diseases. EVs carry molecular signatures reflective of physiological and pathological states, making them a valuable tool for detecting and monitoring a range of gynecological conditions (Figure 1) [18]. Notably, EVs have been implicated in identifying biomarkers associated with infertility, polycystic ovary syndrome (PCOS), endometriosis, and other reproductive disorders [4]. Furthermore, the molecular content of EVs has shown potential in providing insights into pregnancy outcomes, offering a novel approach to understanding maternal–fetal health and disease progression [19].

#### 3.1.1. Follicular Fluid (FF) EVs

The increased number altered profile of FF EVs can predict a natural ovarian aging [20]. PCOS patients had a higher amount of EVs in their follicular fluid than healthy fertile women, and their protein content can regulate granulosa cell activity and steroidogenesis [21,22]. Also, FF EVs contain biomarkers, mostly non-coding RNAs, predicting fertilization failure or low-quality embryos [23,24,25].

#### 3.1.2. Endometrial EVs

Endometrial EVs have been identified not only in endometrial tissue but also in uterine lavage, menstrual blood, and vaginal secretions, and are increasingly recognized for their diagnostic and mechanistic value in infertility research. Studies have shown that uterine lavage-derived small EVs from infertile women contain fewer proteins with antioxidant activity, which are associated with successful embryo implantation, and endometrial receptivity. These EVs also carry small non-coding RNAs implicated in immune regulation, extracellular matrix remodeling, and cell junction formation [26,27]. EVs isolated from menstrual blood serum and those released by menstrual blood-derived stromal cells (MenSCs) from women diagnosed with unexplained infertility have lower amounts of proteins associated with cell adhesion, apoptosis, and oxidative stress [28,29,30]. EVs from secretory and gestational phase endometrial organoids of women with adenomyosis contained miRNAs associated with implantation failure, preeclampsia, and miscarriage [31]. EVs were isolated from endometrial stromal cells or vaginal secretions of women with endometriosis-associated infertility and healthy controls. Small RNA sequencing analysis revealed that the primary targets of differentially expressed miRNAs were involved in signaling pathways such as MAPK, ErbB, AMPK, Wnt, and FoxO, as well as processes including endocytosis, EGFR tyrosine kinase inhibitor resistance, and adherent junction regulation. They also decreased intracellular Ca2^+^ and motility parameters (total motility, progressive motility, and linear velocity) of human sperm in vitro and reduced conception rate [32,33]. Endometrial cell EVs, isolated from the endometrium tissue of women with recurrent implantation failure, reduced embryonic invasion capacity [34].

#### 3.1.3. Placental EVs

During pregnancy, EVs are released into both maternal and fetal circulations, where they facilitate maternal–fetal communication, modulate immune tolerance, and support fetal development by transporting essential nutrients and signaling molecules [5]. Placental EVs have also been implicated in the pathophysiology of pregnancy-related complications such as preeclampsia, gestational diabetes mellitus (GDM), and preterm birth, and are emerging as potential biomarkers for the early diagnosis of these conditions [35,36,37]. Elevated levels of placental EVs have been found in the plasma of women with preeclampsia, with their cargo enriched in inflammatory mediators, factors linked to trophoblast dysfunction and impaired spiral artery remodeling. Similarly, increased numbers of proinflammatory EVs were observed in GDM patients, suggesting a role in systemic inflammation [38]. In cases of preterm birth, reduced levels of PLAP^+^ EVs were detected compared to term pregnancies. These EVs carried molecular signatures associated with enhanced inflammation and suppressed coagulation and complement pathways, contributing to the risk of early delivery [39].

These findings suggest that EVs hold a significant potential as diagnostic tools for various female reproductive disorders; however, their utility as a source of biomarkers requires validation in larger cohorts.

#### 3.1.4. Limitations

However, translation into clinical diagnostics remains limited by heterogeneity in sample types, EV isolation and quantification protocols, and lack of standardized normalization strategies. Many studies are based on relatively small cohorts and require validation in larger, prospective populations. Harmonization efforts aligned with MISEV2023 recommendations will be critical to support reproducibility and clinical implementation.

### 3.2. Therapeutic Application

Recent studies have highlighted the significant therapeutic potential of EVs derived from endometrium, trophoblast cells or placenta in the context of reproductive health [40,41,42]. These EVs, known for their ability to mediate intercellular communication and deliver bioactive molecules, have demonstrated encouraging results in improving fertility outcomes both in vitro and in vivo. Mesenchymal stem/stromal cell (MSC)-derived EVs, depending on their cellular origin, exhibit diverse regenerative properties that contribute to the repair and functional recovery of reproductive tissues, as demonstrated in preclinical studies. For example, the EVs from human endometrial-derived MSCs increased embryo number and quality in aged murine IVF models by reducing the gene expression of oxidative-stress enzymes and increasing the expression of the angiogenic factor VEGF [40]. MenSC-derived EVs increased the number of follicles and live births in a premature ovarian insufficiency murine model [41]. Uterine luminal-derived EVs improved trophoblast cell proliferation and migration in a porcine model [43]. Placental EVs showed very promising data in inducing necrosis in human ovarian and cervical tumor explants and mice ovary tumors [42,44,45].

These findings highlight that EVs and their molecular cargo play a key role in the regulation of the female reproductive system and represent promising tools for the treatment of various reproductive disorders.

## 4. EVs for Ophthalmic Therapeutics

Nanoscale EVs that include exosomes and microvesicles are promising therapeutic vehicles in ocular diseases due to their possession of anti-inflammatory, antiapoptotic, tissue-repairing, neuroprotective, and immunomodulatory properties [46,47]. In ophthalmology, EVs have emerged as promising agents, capable of penetrating biological barriers and immune-privileged tissues. Their unique properties, including biocompatibility and low immunogenicity, make them well-suited for delivering advanced treatments for various eye diseases [46]. According to the World Health Organization (WHO), the leading causes for visual impairment are cataracts, glaucoma, uncorrected refractive errors, age-related macular degeneration, diabetic retinopathy, and among others, corneal opacities affecting more than 2.2 billion people worldwide, leading to a large burden for individuals, their families, and socioeconomic status [48,49]. Due to the limitations of surgical and pharmaceutical therapeutic interventions and their availability depending on the geographical region, a significant unmet need persists for newer and more effective treatment options for ocular diseases [47,50]. The established and potential therapeutic benefits of EVs in the field of eye diseases are enormous and have been highlighted in numerous studies across a wide range of ophthalmic conditions including corneal injury, dry eye syndrome, glaucoma, diabetic retinopathy (DR), age-related macular degeneration (AMD), retinal degenerative disorders such as retinitis pigmentosa (RP), and ocular inflammatory conditions [47].

Although the eye is an immune-privileged organ compared to others, many immune-mediated diseases can harm anterior and posterior segment ocular tissues, as seen in Sjögren syndrome or autoimmune uveitis [48,51]. EVs can modulate the overactive immune response in these pathologies by promoting the differentiation of macrophages and regulatory T-cells, and by reducing the effector T-lymphocyte and natural killer cell count. Recent data show that EVs can penetrate through biological barriers and possibly also the barriers of the eye (tear film, corneal stromal, and blood–retinal and blood–aqueous barriers) [52]. The blood–ocular barriers are composed of two significant obstacles to overcome: the blood–aqueous barrier (BAB), formed by ciliary epithelium, iris vascular endothelial cells, and Schlemm’s canal endothelium, and the blood–retinal barrier (BRB), shaped of retinal vascular endothelial cells, retinal pigment epithelium (RPE), Bruch’s membrane, and choriocapillaris [52,53]. These barriers block the entrance of immune cells from peripheral blood, creating a unique immunosuppressive environment in the eye. Furthermore, the BRB is largely responsible for the limitation of drug absorption in the posterior segment of the eye [52,53]. EVs can easily transport the drugs through such blood–ocular barriers due to their lipid bilayer membrane and nanosized dimensions, and can persist in the ocular tissues for a long period of time. The major routes of administration for EVs are topical delivery (eye ointments and drops), subconjunctival and transscleral, intravitreal, retrobulbar, and subretinal injections [46,47,49,50]. Studies on animal models and early clinical trials have demonstrated safety and efficacy using EVs delivered via these routes, with reduced risk of immune rejection or tumor formation compared to cell therapies [51].

The origin of EVs has been from the ocular system, circulatory system, and stem cells. They have been found in ocular biofluids (tears, aqueous and vitreous humor) and serum/plasma, which can be easily accessible and serve as an excellent diagnostic biomarker for many ophthalmologic diseases [53]. Extracellular vesicles from ocular fluids are isolated using methods compatible with low sample volumes, including differential ultracentrifugation, precipitation-based kits, ultra-filtration, size-exclusion chromatography, and immunoaffinity approaches, with ultra-centrifugation remaining the most widely used reference technique. EV quantification and characterization are commonly performed using nanoparticle tracking analysis or resistive pulse sensing to determine size and concentration, complemented by microscopy and protein-based assays to confirm vesicle morphology and EV-specific markers, with combined approaches required to ensure reliability in complex ocular samples [53]. However, tear-derived EV profiling can be influenced by tear film variability and pre-analytical factors such as reflex tearing, ocular surface inflammation, topical therapy use, and sample collection technique, which should be considered when interpreting biomarker studies. Ocular tissues, like the cornea and retina, produce and respond to EVs, implicating these vesicles in the pathogenesis and treatment of diseases such as dry eye disease, corneal injuries, diabetic retinopathy, and age-related macular degeneration (AMD) [46,47,50].

### 4.1. Dry Eye Disease (DED)

Dry eye disease (DED) occurs due to tear film instability and ocular surface inflammation, which can result in discomfort and visual disturbance. EVs, especially exosomes, are being actively studied for diagnostic and therapeutic roles in DED [50,54,55]. Derived from MSCs, including those from adipose tissue or umbilical cord sources, growing data evidence has shown that EVs carry anti-inflammatory cytokines, growth factors, and miRNAs that may support epithelial repair and reduce inflammation [56]. Studies involving the topical application of MSC-derived EVs in animal models have indicated improvements in corneal epithelial healing, a reduction in proinflammatory cytokines (such as IL-1β and TNF-α), and increased tear production [57,58]. Wang et al. also found that exosomes from human umbilical cord MSCs decreased inflammation and supported corneal epithelial regeneration in a dry eye mouse model [55]. Li et al. observed that EVs can influence immune cell infiltration in the lacrimal gland and improve tear film stability [59,60]. MSC-derived EVs have shown efficacy in experimental models, leading to significant improvements in tear production and corneal integrity, reduction in epithelial apoptosis, and modulation of the inflammatory response, primarily through pathways such as PD-L1 and NLRP3 [60]. Studies using human adipose-derived stem cell EV eye drops in mice have demonstrated the suppression of NLRP3-mediated inflammation and repair of ocular surface damage [60]. However, clinical applications are currently limited by technical obstacles in EV isolation and delivery, requiring further research for optimization in humans.

### 4.2. Corneal Injury

According to the WHO, corneal damage is responsible for 5.1% of total blindness globally [49,61]. A vast etiology of corneal damage includes infection, trauma, chemical burns, and ocular surgeries, and acquired and inherited ocular diseases lead to corneal inflammation and fibrosis, causing scarring and vision loss [62]. For corneal disorders, including wounds and scarring, EVs accelerate epithelial healing and modulate the local immune microenvironment [63]. Experimental models show that corneal stromal cell-derived EVs can enhance corneal transparency and reduce fibrosis, suggesting broad applications in post-injury and post-surgical recovery [64,65,66,67]. EVs from corneal stromal stem cells (CSSCs) or MSCs deliver growth factors (e.g., TGF-β, HGF) and miRNAs that support cell growth, reduce fibrosis, and adjust immune responses [64]. Animal studies show that EVs speed up healing and lessen haze [64,65]. EVs derived from corneal epithelial cells accelerate epithelial wound healing and stromal regeneration. These vesicles promote re-epithelialization, inhibit inflammation, decrease fibrosis, and restore corneal transparency following injury [63,64,65,68]. Advanced delivery systems, like biocompatible hydrogels, are being developed to achieve sustained and on-demand EV release, overcoming rapid ocular clearance and improving therapeutic efficacy [69]. Such systems mirror natural tissue remodeling processes and can significantly improve corneal healing outcomes [64,69].

### 4.3. Glaucoma

In glaucomatous optic neuropathy, characterized by progressive retinal ganglion cell (RGC) death and axonal degeneration, MSC-derived EVs and retinal pigment epithelium (RPE)-derived EVs exert neuroprotective and neuroregenerative effects [46,70,71]. Mechanistically, EVs enrich neurotrophic factors such as brain-derived neurotrophic factor (BDNF) and glial cell line-derived neurotrophic factor (GDNF), and convey protective microRNAs (e.g., miR-17-92 cluster), which collectively enhance RGC survival by modulating apoptotic signaling pathways and mitochondrial function [70,71].

Experimental glaucoma models reveal that intravitreal administration of MSC-EVs reduces retinal inflammation by suppressing microglial activation via EV-contained anti-inflammatory cytokines and miRNAs like miR-124 [70]. Additionally, EV treatment promotes axonal outgrowth and synaptic repair, key for preserving visual pathways impaired in glaucoma. These findings are encouraging for addressing the unmet need for neuroprotective therapies complementing intraocular pressure management [71].

### 4.4. Age-Related Macular Degeneration (AMD)

AMD pathogenesis involves the degeneration of RPE cells and aberrant choroidal neovascularization driven by chronic inflammation and oxidative stress. EVs derived from retinal or MSC sources have demonstrated multifaceted therapeutic effects based on their cargo of antioxidative enzymes, immunomodulatory factors, and angiogenesis regulators [71,72]. They modulate the retinal microenvironment by downregulating VEGF expression via the delivery of specific microRNAs (e.g., miR-15a, miR-27b), inhibiting pathological neovascular sprouting that leads to vision loss [72]. MSC-EVs specifically reduce leukocyte adhesion and proinflammatory cytokine secretion by repressing NF-κB and ICAM-1 pathways within retinal endothelial cells and microglia, thereby limiting capillary dropout and vascular leakage [72]. Additionally, the EV-mediated transfer of miR-150 and miR-221 stabilizes angiogenesis by promoting pericyte function and endothelial cell integrity, thus preventing aberrant neovascular tuft formation [72].

Intravitreal injections of EVs in diabetic animal models have demonstrated the restoration of blood–retinal barrier integrity, reduced oxidative stress, and functional improvement of retinal electrophysiology. These multifactorial benefits position EVs as promising candidates for novel DR therapies that address both vascular and neuroinflammatory components [71,72].

Furthermore, RPE-derived EVs contain complement regulators and antiapoptotic molecules that mitigate complement-mediated cytotoxicity and enhance retinal cell survival, concomitantly reducing inflammasome activation [70,71,72]. Engineered EVs capable of enhanced mitochondrial protection and oxidative stress resistance further highlight the translational potential of EVs as adjuvant therapies to current anti-VEGF injections, aiming for sustained retinal preservation [50,72].

### 4.5. Diabetic Retinopathy (DR)

In DR, hyperglycemia-induced retinal microvascular damage and inflammatory cascades culminate in vascular leakage, ischemia, and pathological angiogenesis. EVs from endothelial cells, MSCs, and pericytes have emerged as critical modulators of these processes through the delivery of regulatory microRNAs (e.g., miR-126, miR-15a-5p), proteins (angiopoietins, tissue inhibitors of metalloproteinases), and anti-inflammatory cytokines [50,72].

Altogether, extracellular vesicles play a unique and multifaceted role in ophthalmology, functioning as disease-specific biomarkers, intrinsic therapeutic agents, and advanced drug delivery systems. EV biomarkers provide accessible molecular insights into ocular pathophysiology through their cargo in ocular fluids and circulation, supporting diagnosis, prognosis, and treatment monitoring. EV therapeutics harness the natural anti-inflammatory, neuroprotective, and tissue-repair capacities of vesicles to directly modulate disease processes, while EVs as drug carriers extend this potential by enabling targeted, sustained delivery of exogenous therapeutics across ocular barriers. These complementary applications underscore the growing translational relevance of EVs and position them as a versatile platform for both precision diagnostics and next-generation therapies in ophthalmology.

## 5. EVs in Otorhinolaryngology

Extracellular vesicles have a wide range of potential applications in otorhinolaryngology. Preclinical animal and in vitro studies provide the strongest evidence for EV applications in ORL, while human data remain limited to early feasibility and safety studies, highlighting the need for clinical validation to confirm therapeutic efficacy; a concise overview of these applications can be seen in Table 1.

EVs, particularly exosomes, emerge as practical cell-free therapeutic tools because they carry functional RNAs, proteins, and lipids, and can be cryopreserved and delivered through established ORL routes, including intranasal and intratympanic administration. Unlike living cell grafts, they impose lower immunological and logistical burdens [94]. In parallel, EV cargo in accessible ORL biofluids—especially saliva and nasal lavage—provides non-invasive biomarker readouts for detection, endotyping, and treatment monitoring, aligning with the routes used for therapies [73,95].

### 5.1. Sensorineural Hearing Loss (SNHL)

Early EV-based monitoring emerges in otologic disease, for example, keratinocyte-derived exosomes from cholesteatoma upregulate RANKL via exosomal miR-17, marking osteolytic activity that complements imaging and staging [74]. In sensorineural hearing loss (SNHL) caused by noise, aging, or ototoxic agents, regenerative capacity is minimal, and preclinical studies show that MSC-derived EVs can counteract these injury-driven mechanisms by protecting cochlear hair cells, reducing oxidative stress, and promoting spiral ganglion neuron survival [75,76]. In cisplatin-injured mice, umbilical cord MSC exosomes delivered systemically or intratympanic improved auditory thresholds, with local dosing proving superior [77]. Clinically, a first-in-human case report documented the safe intracochlear application of human stromal cell EVs during cochlear implantation, suggesting perioperative EV dosing could mitigate surgical trauma [78]. The heat shock preconditioning of MSCs further enriched EVs in HSP70, which attenuated cisplatin ototoxicity in animal models [79].

### 5.2. Laryngeal and Vocal Fold Pathologies

Laryngeal and vocal fold pathologies are another major target. Chronic vocal fold scarring leads to fibroblast-to-myofibroblast transition and extracellular matrix (ECM) stiffening. Although direct clinical EV trials are pending, rabbit models show that MSC-loaded PEG-fibrin hydrogels restore lamina propria structure and biomechanical properties, highlighting the potential of EV-enriched biomaterials for scar remodeling [96]. Epidermal stem cell EVs have been shown to reverse myofibroblast activation in dermal fibrosis, offering mechanistic support for vocal fold regeneration [97].

### 5.3. Airway Inflammatory Diseases

In airway inflammatory diseases such as chronic rhinosinusitis (CRS) and allergic rhinitis (AR), EVs act as immune regulators. Additionally, nasal lavage-derived EV miRNA and protein signatures can distinguish between different CRS endotypes and provide readouts of epithelial barrier integrity and type 2 helper T-cell (Th2)-driven inflammation [82,95]. From a therapeutic standpoint, epithelial and immune cell EVs can carry miRNAs (e.g., miR-21, miR-155) that amplify Th2 responses, whereas therapeutic MSC-EVs counteract these pathways and reduce inflammation [80,83]. In animal models, intranasal adipose-derived stromal cell (ASC)-EVs reduced eosinophilia and promoted regulatory T-cell (Treg) responses, while MSC-EVs restored epithelial integrity [84,85]. Engineered modifications such as aptamer binding further enhanced mucosal retention and efficacy [83]. Importantly, the intranasal route provides not only sinonasal targeting with minimal systemic exposure but also nose-to-brain access, relevant for comorbid neurological diseases [81,98].

### 5.4. Head and Neck Cancer

In head and neck squamous cell carcinoma (HNSCC) and nasopharyngeal carcinoma (NPC), tumor-derived EVs promote invasion, angiogenesis, and immune evasion [86,89]. These pathogenic roles are reflected in their diagnostic potential, where in HNSCC, saliva- and plasma-derived exosomes carrying PD-L1, CD44v3, or composite miRNA panels enable non-invasive detection, risk stratification, and disease monitoring [99,100]. In NPC, circulating exosomal Epstein–Barr virus (EBV) miR-BARTs and LMP1 serve as tumor-specific markers correlated with burden and radioresistance, supporting non-invasive surveillance [89]. Furthermore, EVs can serve as both therapeutic targets and delivery platforms [86]. Engineered exosomes delivering miR-34a or siRNA against LCP1 suppressed proliferation, migration, and xenograft growth in oral squamous cell carcinoma (OSCC) models [87,88]. In NPC, where EBV-linked EV cargo such as LMP1 and viral miRNAs contribute to immune suppression and therapy resistance, antagomiRs (chemically engineered inhibitors of specific microRNAs) packaged in engineered EVs successfully blocked EBV-miRNA activity, reducing angiogenesis and invasion in preclinical systems [90,91].

### 5.5. Surgical Complications

Finally, in surgical complications such as pharyngocutaneous fistula (PCF) after laryngectomy, exosomes have been proposed as proangiogenic, ECM-modulating adjuncts to accelerate wound closure. Experimental evidence shows EVs stimulate fibroblast proliferation, angiogenesis, and matrix deposition, making them promising complements to flaps and advanced dressings in irradiated fields. To complement these interventions, conceptual evidence suggests that wound fluid EV cargo linked to angiogenesis and ECM remodeling could serve as adjunctive indicators of healing trajectories in irradiated fields, although specific validated EV biomarkers for PCF monitoring have not yet been established [92].

### 5.6. Other ORL Applications of EVs

Beyond disease modification, EVs also excel as drug delivery systems tailored to ORL anatomy [81,94]. Intratympanic delivery allows high local inner ear concentrations with minimal systemic exposure, while intranasal administration targets sinonasal tissues and can be exploited for treating central neural system (CNS) co-morbidities [81,94,98]. Practical payloads include heat shock proteins, small molecule drugs, and tumor suppressive RNAs [79,81]. Clinically, feasibility is supported by a study on autologous ASC exosomes as an adjunctive therapy for periodontitis (NCT04270006) [93].

### 5.7. Broader Relevance of ORL EV Studies and Routes

ORL EV research operates as a translational delivery platform, whose principles extend to neurology, oncology, and mucosal immunology beyond the head and neck field [81,94,101]. The intranasal administration—via olfactory and trigeminal pathways—enables repeatable, needle-free dosing to the airway mucosa with potential nose-to-brain transport, offering a practical template for regional and central delivery without systemic burden [81,98]. Rodent studies show that intranasally administered EVs reach the olfactory bulb and deeper brain structures within hours and modulate neuroinflammation, supporting their use for CNS-adjacent indications that benefit from rapid, regional exposure [98].

Since this route is accessible and immunologically rich, ORL airway studies serve as testbeds for mucosal immunoengineering, demonstrating MSC-derived and engineered EVs can rebalance Th2/Treg responses and restore nasal epithelial barrier programs in allergic rhinitis models [80,83,85]. Furthermore, converging preclinical evidence points to translational implications for asthma and viral upper respiratory infections and provides a rationale for exosome-based vaccine adjuvants [80,102].

The intratympanic route enables compartment-targeted microdosing in the cochlea, maximizing local perilymph exposure while limiting systemic spillover. Taken together, ORL routes offer controllable exposure profiles providing tunable parameters, such as dose, frequency, and formulation, to adapt EV delivery to diverse tissues [81,94]. ORL oncology models illustrate EVs as both targets, disrupting tumor EV biogenesis, and tools, using engineered EVs as RNA carriers, a concept transferable to other epithelial malignancies sharing hypoxic niches and immune-evasive EV crosstalk [86,87,88,90,91]. These principles extend beyond ORL cancers, where exosomes are being developed as both therapeutic agents and delivery vehicles, including anti-KRAS and stromal-reprogramming strategies in pancreatic ductal adenocarcinoma (PDAC), miRNA-loaded exosomes in triple-negative breast cancer (TNBC), and EV-based chemosensitization and immune modulation in colorectal cancer (CRC) [103,104,105].

Methodologically, ORL-proximal work highlights how the adoption of rigorous standards (MISEV2023) and fit-for-purpose analytics strengthens translational reliability, from experimental design to reporting [106]. Salivary EV studies show that isolation choices and particle readouts, e.g., nanoparticle tracking analysis (NTA), a technique that measures EV size and concentration based on Brownian motion, materially influence assay robustness and biomarker detection, providing a blueprint for biofluid assay development in other fields [107,108]. Together with GMP-compliant manufacturing frameworks and strategic development guidance, these advances reinforce the practicality and clinical readiness of ORL-derived EV delivery strategies across diverse indications [101,109].

### 5.8. Challenges and Perspectives

Despite the exciting promise of exosome therapeutics and diagnostics in ORL diseases, several challenges must be solved before these tools can be used in everyday clinical practice. A key issue is scientific rigor: the MISEV2023 guidelines stress that researchers should carefully describe exosome isolation, test them with more than one method, and prove that any observed effects really come from the exosomes themselves. Without this, it is difficult to decide the right dose or to compare results between different studies [106]. Isolation variability is clear in salivary EV work, where ultracentrifugation, precipitation, and immunocapture yield different proteomes, highlighting the need for standardized protocols [107]. Even basic counting tools such as NTA can give variable results depending on instrument configuration and analysis parameters. Fluorescence NTA improves specificity, but potency still requires orthogonal validation such as cargo copy number quantification or mechanism-linked bioassays [108].

On manufacturing and formulation, scalable GMP production remains an issue. Robust release criteria must capture identity, mechanism-linked potency, sterility, and stability, and batches must remain comparable across donors and process changes [101,109]. Route-specific bioavailability is also important. For intranasal delivery, many vesicles are lost because they are cleared by mucus, broken down by enzymes, or unevenly deposited. Strategies like adding mucoadhesive coatings or special surface ligands can help reduce these losses [81,83]. For the inner ear, challenges include crossing local barriers and understanding fluid movement, but studies with intratympanic injections and even a first-in-human trial show that this route is feasible [77,78,94].

Additionally, exosomes are a double-edged sword: the same vesicles that provide diagnostic information can also drive disease. In HNSCC, for example, exosomes enriched in PD-L1 suppress T-cell activity and help tumors evade immunity [110]. This dual role makes them attractive to therapeutic targets but also risky, since healthy cells rely on exosomes for normal communication. In immunotherapy, blocking tumor-derived exosomes may boost immune responses, yet broad suppression could also remove beneficial signals, such as those involved in tissue repair. Clinical evidence in ORL is still very limited. So far, it includes a first-in-human intracochlear application and a few small trials in dental, oncology, and other fields, which are encouraging but not enough to prove overall efficacy [78,93,111]. The next step should be practical early-phase studies with clear, route-specific endpoints and manufacturing plans that ensure product consistency [78,101,109].

In oncology, two strategies bring risks: blocking tumor exosomes or using them as delivery vehicles. Both can cause off-target effects, immune changes, or unwanted uptake, so development must include the careful tracking of biodistribution, safety testing, and clear definitions of on-target versus off-target effects [86,87,88,90,91,101]. Finally, determining EV dosage remains unresolved: particle counts, protein levels, RNA copies, and bioactivity are not interchangeable, so a common standard is needed for rational dosing and comparison across studies [106,108].

The main pitfalls of EV-based applications in ORL arise from local delivery constraints, mucosal immune interactions, and strict manufacturing requirements. Intranasal and intratympanic routes face clearance, enzymatic degradation, and uneven deposition that limit bioavailability, while immunologically active mucosa may trigger unintended responses with repeated or tumor-derived EVs. Furthermore, stringent sterility, purity, and GMP standards near sensitive sites like the inner ear and central nervous system necessitate careful validation to ensure safe and effective clinical translation.

## 6. EVs in Urology

### 6.1. Exosomes and Chronic Bladder Pain Syndrome

Interstitial cystitis/chronic bladder pain syndrome (IC/CBPS) is defined as a myriad of lower urinary tract symptoms including dysuria, frequency, urgency, nocturia, and bladder pain or discomfort, which can be expanded to other pelvic areas, without detectable urological cause such as bladder infection, calculi, or tumor, causing significant impairment of life quality. The prevalence of this syndrome varies from 0.01% to 6.5%, and it is five times more common in women. It is hypothesized that inflammation following infection, autoimmune disorder, mechanical lesion, or other noxious factors underlies this pathophysiology. This inflammation may lead to a defective glycosaminoglycan (GAG) layer, impaired urothelium permeability, activation of mast cells, neurogenic inflammation, synaptic and neural plasticity, and ultrastructural changes such as fibrosis and loss of muscle fibers [112]. Pain also stimulates reflexive pelvic floor contraction, causing relative bladder outlet obstruction (BOO), which is clinically apparent as a poor stream, straining, and a sensation of incomplete emptying [113]. Additionally, patients suffering from interstitial cystitis experience sleep disturbance, chronic fatigue, sexual and bowel dysfunction, and anxiety [112]. The therapeutic objective is the absence or at least a reduction in symptoms and an enhancement of the quality of life (QoL) of patients. Guideline recommendations include a stepwise approach, starting with behavioral therapy and modification of life habits, followed by oral medication and instillation of bladder cocktails, and progressing to surgical interventions if needed. As a result of the suboptimal treatment options for IC/CBPS, alternative treatment modalities are emerging, such as stem cell and monoclonal T antibody therapy, as well as exosome application [112]. MSCs showed therapeutic potential in IC/CBPS through migration to the bladder tissue, followed by differentiation into target bladder cell lineages. Simultaneously, MSCs attenuate mast cell infiltration and apoptosis, suppress inflammatory response, reduce extracellular matrix deposition, and promote structural and functional tissue regeneration [114]. Some in vitro studies have demonstrated that MSCs secrete various cytokines with immunomodulatory, anti-inflammatory, antiapoptotic, and proangiogenic properties, resulting in the regeneration of the urinary bladder [115]. Notably, most mechanistic and therapeutic evidence for exosomes in IC/CBPS is currently preclinical (in vitro and animal models), and further well-designed human studies will be necessary to confirm clinical efficacy and safety. For instance, Rubini et al. demonstrated that MSCs released exosomes harboring miRNAs that can induce regenerative processes, such as cell proliferation, immune modulation, angiogenesis, and anti-inflammatory responses, in a feline model. Key miRNAs identified include fca-miR-221, fca-let-7f-5p, fca-miR-337-5p, fca-miR-542-5p, fca-miR-24-3p, fca-miR-205, and fca-miR-23a, which are responsible for the promotion of proliferative, angiogenic, differentiation, and regenerative mechanisms [116]. Experiments in RAG1-deficient mice demonstrated that exosomes release mitogens, namely basic fibroblast growth factor (bFGF), leading to urothelial cell proliferation [117]. Another animal study elucidated that exosomal miR-9 mitigates neuroinflammation and bladder pain by impeding the TLR4/NF-κB/NLRP3 signaling pathway [118]. Sanchez et al. demonstrated that the increased expression of exosomal miRNAs, including miR-449b, miR-500, miR-328, and miR-320, downregulates the expression of the neurokinin 1 receptor (NK1 R), a well-known neuromodulatory factor involved in IC/CBPS pathogenesis. The authors hypothesize that miRNA elevation is a consequence of prolonged agonist exposure and might therefore represent a secondary adaptive mechanism, allowing cells to cope with continuously activated receptor signaling by reducing the response to chronic pain overstimulation [119]. Maternally expressed gene 3 (MEG3) is an exosomal molecule that has been shown to contribute to IC/CBPS pathogenesis by inducing the downregulation of miR-19a-3p expression while upregulating TLR7 expression. According to their findings, Li et al. suggested that urinary MEG3 can be utilized as a biomarker for IC diagnosis [120].

In conclusion, MSC-derived exosomes carry beneficial components, including miRNAs, which could potentially be harnessed for the treatment of IC/CBPS. Some of these elements possess immunomodulatory, anti-inflammatory, antiapoptotic, and proangiogenic properties, resulting in the regeneration of the urinary bladder, making them an attractive tool for regulating immune cell function in IC patients. Nevertheless, further investigation into this subject is mandatory.

### 6.2. Exosomes and Urinary Tract Infections

Urinary tract infections (UTIs) are considered a burdensome diagnosis due to their high prevalence and recurrence [121]. The clinical signs and symptoms vary depending on the loci of the involvement of the urinary tract, thus classifying UTIs as a localized or systemic infection. Localized UTIs usually present a group of symptoms that include dysuria, nocturia, frequency, urgency, and suprapubic pain, without systemic signs and symptoms. Systemic UTIs are characterized by the occurrence of systemic signs and symptoms, primarily represented by chills, fever, and flank or pelvic pain, which raises the suspicion of conditions such as pyelonephritis and prostatitis. Risk factors may complicate the course and should be identified early; some of these factors pertain directly to patients themselves, such as young or advanced age, frailty, and compromised immune status. Male patients with prostatic involvement and female patients during pregnancy or with pelvic organ prolapse are particularly at higher risk. Impaired urination caused by anatomical, neurological, or functional abnormalities of the urinary tract can also predispose to UTIs due to the significant post-void residue. Additional contributors include a history of prior antibiotic use, which may select for resistant organisms, and the presence of urinary stones or urinary obstruction, both of which can create a favorable environment for infection. Indwelling urinary catheters and recent urological procedures further increase the likelihood of infection by facilitating bacterial entry or disrupting mucosal defenses [122]. Antibiotics are a standard treatment for UTIs, and although this therapy is usually successful, UTIs still pose a health hazard to some vulnerable categories, such as frail and immunocompromised patients and pregnant women. Another factor complicating antimicrobial therapy is the development of multiple bacterial resistance. This raises the question of the necessity for new treatment strategies. MSC-derived exosomes have demonstrated a significant therapeutic effect in treating infectious diseases, aiming to eliminate pathogens, diminish antimicrobial resistance, and enhance tissue regeneration. This effect can be achieved through several mechanisms, such as the activation of phagocytes or production of molecules holding antimicrobial properties [123].

The most common bacterial causative agent is uropathogenic Escherichia coli (UPEC), a member of the Enterobacteriaceae family of coliform bacteria. Consequently, much research has centered in elucidating the pathophysiological mechanisms of UPEC-induced UTIs. It is hypothesized that UPEC causes dysfunction of the bladder urothelial barrier, exposing the lamina propria of the bladder to urinary content, resulting in the activation of mast cells and exacerbated inflammation. One possible mechanism involves UPEC infection-induced pyroptosis of bladder urothelial cells, resulting in the exosomal release of IL-1 and IL-18 that subsequently activate mast cells. Activated mast cells secrete tryptase, which induces protease-activated receptor 2 (PAR2), resulting in the disruption of bladder urothelial barrier function and increased bacterial intravasation, thereby reinforcing the vicious cycle of the UPEC UTI [124]. Supporting this mechanism, an in vitro study conducted by Wang et al. demonstrated that UPEC infection stimulates the secretion of large quantities of exosomes from bladder epithelial cells, referred to as MB49 U Exo, meaning exosomes derived from UPEC-infected MB49 cells. These exosomes are further internalized by macrophages, promoting the production of proinflammatory cytokines, particularly TNF-alpha, and reducing the phagocytic activity of macrophages by suppressing phagocytosis-related gene expression, while increasing apoptosis. These proinflammatory effects were mediated by exosomal miR-18a-5p, which promoted TNF-alpha expression by suppressing PTEN and activating the MAPK/JNK pathway. In other words, bladder inflammation can be attenuated by inhibiting exosome release, as demonstrated in a murine UTI model using GW4869. According to these findings, it can be speculated that one therapeutic strategy for treating UTIs may be the inhibition of exosome-mediated TNF-alpha expression [125]. In a murine model of UTI caused by UPEC, urinary exosomes were enriched with lactoferrin, an iron-binding glycoprotein released by bladder epithelial cells during infection. Exogenous administration of human lactoferrin (hLf) reduced UPEC adherence, boosted neutrophil antimicrobial activities, and significantly decreased bacterial load and neutrophil infiltration in the bladder, highlighting lactoferrin’s therapeutic potential as an innate immune modulator against UTI [126]. Proteomic analysis by Hiemstra and his coworkers demonstrated that human urinary exosomes contain innate immune proteins with antimicrobial activity, as well as the ability to inhibit the growth of Escherichia coli [127].

Asymptomatic bacteriuria (ABU) is another problematic clinical “pebble in the shoe”. It should not be treated unless it is present in vulnerable population categories, such as pregnant women. Still, the clear distinction of ABU from UTI is sometimes blurred, especially in old and institutionalized individuals, and there is no clinically available marker for an accurate differential diagnosis. Mizutani et al. hypothesized that the molecular signature of urinary exosomes may vary between UTI and ABU patients, demonstrating that levels of the intracellular signaling molecules Akt and ERK, along with the transcription factor NF-kappa B, were elevated in exosomes derived from THP-1 and SV-HUC-1 cells co-cultured with Escherichia coli. Additionally, exosomal Akt and CD9 were significantly diminished in the urine of UTI patients, suggesting that they could be valuable markers for the differential diagnosis of UTI and ABU [128].

## 7. EVs for Cancer Immunotherapy

**Figure 2:**
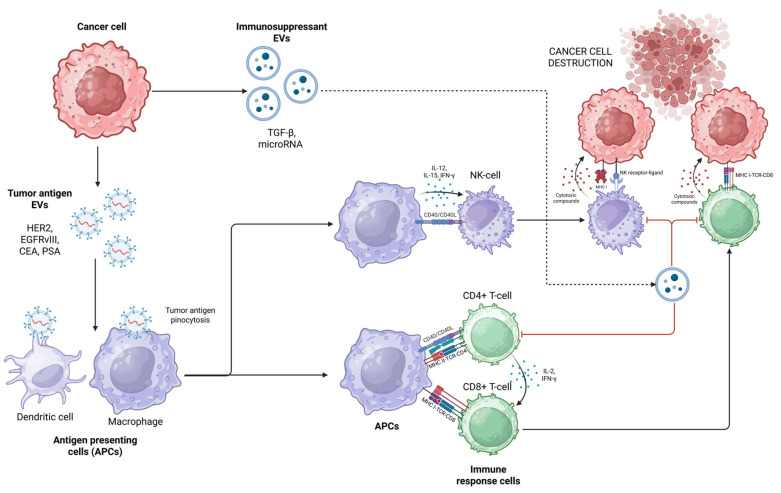
EV-mediated interactions between cancer cells and immune cells in the tumor microenvironment (created with Biorender.com).

Extracellular vesicles, particularly exosomes, play a crucial role in the interactions between cancer cells and the immune system, predominantly through the transport and presentation of tumor antigens (Figure 2).

Cancer cells release extracellular vesicles into their microenvironment, which may contain polypeptides, DNA fragments, RNA fragments, and specific tumor antigens [129]. These antigens may be present either on the surface of the vesicle or inside its lumen. Tumor antigens commonly found on the surface of extracellular vesicles include HER2 (present in breast cancer), EGFR and mutated EGFRvIII (present in glioblastoma), CEA, PSA, and others [130,131]. After release, these vesicles interact with antigen-presenting cells, which then process and express the tumor antigens either via the MHC class I or MHC class II complex. This triggers an antitumor immune response, followed by parallel signaling mechanisms. It is also worth mentioning that tumor EVs may contain immunosuppressors, such as TGF-β or microRNAs that inhibit T-cell and NK-cell function, thereby enabling cancer cells to evade the immune system rather than triggering a proinflammatory response [132]. Because of these immunomodulatory properties, EVs are continuously being explored as cancer vaccine components, neoantigen carriers, and therapeutic response indicators detected in liquid biopsies [133].

As a result of their transport capabilities, as well as high stability and low immunogenicity in the human organism, EVs have become potential vectors for the delivery of therapeutic agents in cancer immunotherapy. Extracellular vesicles can be loaded with therapeutic agents using two main strategies [134]. Endogenous loading involves the genetic or pharmacological manipulation of EV-producing cells. For example, a cell transfected with a plasmid encoding interleukin-12 will produce and deliver vesicles containing this cytokine, thus stimulating the antitumor immune response. Exogenous loading refers to the insertion of therapeutic agents into the vesicles themselves, either by electroporation, lipid incubation, or saponin-based loading [135]. In addition to their cargo, the molecules expressed on the EV membrane can also be modified. This allows engineering vesicles to express tumor-specific ligands, such as EGFR or HER2 [136].

So far, several preclinical and clinical studies have investigated the potential of extracellular vesicles as a drug delivery system in cancer treatment. The review article published by Yao Y et al. highlights several clinical trials using dendritic cell-derived exosomes to treat non-small cell lung cancer, melanoma, and colorectal cancer [137]. Results showed an altered immune system response, primarily regarding a partial shift in NK-cell activity. Changes in T-cell responses were also detected, although they did not necessarily lead to a visible shift in the clinical course of the disease. Another article, published by Nikfarjam S et al., also discusses the potential and safety of dendritic cell-derived exosomes as cancer immunotherapy [138]. The article highlights the feasibility and safety of implementing exosomes through several phase I and phase II clinical trials. The primary mechanism of stimulating antitumor activity is precisely via triggering T-cell and NK-cell response. However, the authors also highlight that in advanced malignant disease, exosome vaccines may correlate with low clinical impact. A recent paper published by Zhao et al. discusses the potential use of chimeric antigen receptor (CAR) immune cells, or more specifically, the use of exosomes derived from CAR cells [139]. This novel approach builds on the previously established knowledge from CAR-based therapy, such as CAR-T or CAR-NK cell therapy. However, CAR exosomes possess several advantages over CAR cells, such as easier penetration of solid tumor membranes, easier crossing of blood vessel barriers, smaller size, and lower risk of toxic side effects such as immune effector cell-associated neurotoxicity syndrome (ICANS).

Although the scientific literature has shown promising results so far, there are still challenges in using extracellular vesicles as cancer immunotherapy. One such challenge is the procurement of the vesicles themselves. Firstly, in the context of cancer treatment, the isolation of cancer-derived exosomes might negatively impact the desired outcome as these exosomes may carry immunosuppressant compounds which help the tumor evade the immune system. Additionally, the method of isolation is complex by itself and lacks standardization in the field [140]. Another hurdle for translating EV-based cancer therapy into clinical practice is the still relatively small amount of real-world in vivo data, as most studies in the literature are predominantly in vitro [141].

Nevertheless, extracellular vesicles show great promise as immune response modulators and drug delivery platforms in the future of precision oncology. Through combining cell-based therapies and genetic engineering, this innovative and highly effective treatment modality may see clinical implementation in the not-so-distant future.

### Clinical Landscape

EVs, particularly dendritic cell-derived exosomes, are currently being evaluated in phase I and II clinical trials for cancer immunotherapy, including non-small cell lung cancer, melanoma, and colorectal cancer, with primary endpoints assessing immune activation such as T-cell and NK-cell responses, and secondary endpoints evaluating safety and feasibility [137,138]. Early results suggest that EV administration is generally well tolerated, with no severe immune-related adverse events, although clinical efficacy in advanced malignancies remains modest [137,138]. Emerging approaches, including CAR-derived exosomes, aim to enhance tumor penetration, improve antitumor immune responses, and reduce systemic toxicity, representing a next-generation strategy in precision oncology [139]. Despite this progress, challenges remain in EV isolation, large-scale production, and standardization, which must be addressed to translate promising preclinical findings into robust clinical outcomes [140].

## 8. EVs in Orthopedic Surgery

Trauma-related injuries such as bone fractures, ligament tears, and post-surgical complications often require biological support for efficient tissue regeneration. Bone healing is a complex process involving inflammation, osteogenic differentiation, and vascular remodeling, as these issues often lead to degenerative diseases such as osteoarthritis (OA) [142,143]. There are numerous studies suggesting EVs as a potential therapeutic approach after traumas to enhance bone regeneration by promoting osteogenesis and angiogenesis through the delivery of proregenerative microRNAs (miRNAs) such as miR-21, miR-26a, and miR-196a [144,145]. Moreover, EVs have demonstrated potential in preventing or mitigating post-traumatic complications such as delayed union and infection. In preclinical models, MSC-derived EVs improved fracture healing (osteogenesis and angiogenesis) outcomes by enhancing callus formation through activation of the BMP-2/Smad1/RUNX2 and HIF-1α/VEGF signaling pathways and modulating the local immune environment [146]. Additionally, EVs can be engineered to deliver antimicrobial agents or targeted RNAs to prevent infections associated with orthopedic implants. For example, EVs functionalized with cationic antimicrobial peptides have demonstrated enhanced stability and efficacy in preclinical sepsis models. Strategies such as surface coating, cargo replacement, and stimuli-responsive functionalization further illustrate the versatility of EV engineering for targeted delivery [147,148].

OA, a degenerative joint disease marked by cartilage degradation, inflammation, and subchondral bone remodeling, also lacks effective disease-modifying treatments. In recent years, EVs, particularly those derived from MSCs, have gained attention as an OA-therapy due to their chondroprotective and anti-inflammatory effects. Bone marrow-derived mesenchymal stem cell extracellular vesicles (BMMSC-EVs) have been shown to stimulate chondrocyte proliferation, extracellular matrix synthesis (e.g., collagen II, aggrecan), inhibit apoptosis, and suppress catabolic factors such as MMP-13 and ADAMTS-5 [149,150]. Also, intra-articular injection of MSC-derived EVs in OA models leads to improved cartilage structure and reduced synovial inflammation. Notably, miRNAs such as miR-140-5p and miR-92a-3p, delivered by EVs, contribute to matrix synthesis (COL2A1, COMP) and anti-inflammatory/chondrogenic signaling pathways [151,152].

Clinical translation, however, remains in the early stages. While preliminary clinical trials have indicated good safety profiles, efficacy data remain limited [153]. The heterogeneity of EV preparations and variability in OA severity across patients complicate outcome interpretation and regulatory approval. For instance, a randomized, triple-blind, placebo-controlled clinical trial investigating placental MSC-derived EVs for grade 2–3 knee OA demonstrated that a single intra-articular injection was safe, showing no systemic complications or significant local reactions, but also no improvement in symptoms or MRI outcomes compared to the placebo [153]. On the other hand, one of the recent reviews in EV application for OA highlighted the disease-modifying potential of the MSC-derived secretome and EVs but also underscored the critical need to reduce protocol variability across EV isolation, characterization, and application methods for successful clinical translation [154].

Injuries of soft tissues, like muscle and tendon, are frequent in sports and orthopedic trauma and often result in prolonged recovery and fibrosis. EVs offer a cell-free regenerative approach by modulating satellite cell activity, fibroblast phenotype, and inflammation. For instance, EVs from myoblasts have been shown to enhance myogenic differentiation and reduce fibrosis in murine models of muscle injury by promoting satellite cell proliferation, increased expression of regenerative markers (e.g., Pax7 and PCNA), and reduced fibrotic factors such as collagen-1 and α-SMA, ultimately accelerating injury repair [155]. It was also shown that EVs from adipose tissue MSCs support muscle repair by delivering growth factors like IGF-1, HGF, FGF-2, VEGF, PDGF-AA, and IL-6, and proregenerative miRNAs including miR-1, miR-133, miR-206, miR-125b, miR-494, and miR-601; these factors collectively enhance satellite cell activity, angiogenesis, and reduce fibrosis in models of muscle injury [156]. In tendon repair, EVs may contribute to tenocyte proliferation, ECM organization, and modulation of proinflammatory cytokines. EVs derived from adipose tissue MSCs deliver specific miRNAs, such as miR-29a and miR-221, which contribute to enhanced tendon healing, improved biomechanical strength, and favorable ECM composition, notably increasing levels of decorin and biglycan [157]. In rat tendon injuries, BMMSC-EVs led to better-aligned collagen fibers, as they elevated collagen I relative to collagen III, improving ECM organization toward a more functional tendon-like structure [158]. However, the avascular and fibrous nature of tendons presents challenges for EV-based delivery.

Beyond traditional intra-articular injections, novel administration routes and engineering strategies for customizing EV uptake are being explored to overcome these barriers, including scaffold-based EV delivery systems and ultrasound-mediated enhancement [159]. The therapeutic efficacy of EVs in orthopedic applications is significantly influenced by delivery optimization strategies that address rapid clearance and limited tissue penetration. Hydrogel-based delivery systems have emerged as promising carriers for sustained EV release, overcoming the limitations of rapid degradation and short maintenance during administration [160,161]. Recent advances include injectable and bioactive hydrogels loaded with hypoxic EVs for accelerated bone regeneration and composite hydrogel delivery systems for treating periprosthetic osteolysis [162,163]. Surface modification strategies, including membrane engineering techniques, have been developed to enhance EV targeting capacity toward specific cell types, addressing the natural biodistribution limitations where EVs are mainly concentrated in the liver and spleen [164,165]. These multifaceted approaches to delivery optimization represent crucial steps toward translating EV-based therapies from preclinical models to effective clinical treatments.

**Figure 3:**
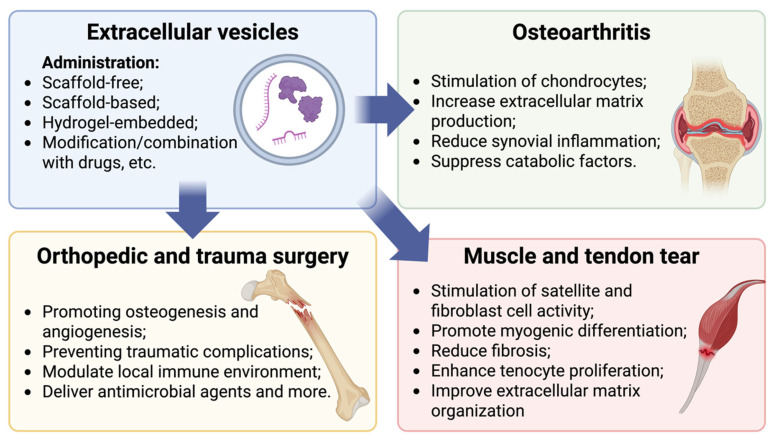
Positive effects of extracellular vesicle application for orthopedic and trauma surgery, osteoarthritis, and muscle/tendon tear (created with Biorender.com).

Thus, EVs represent a promising therapeutic tool in orthopedic and trauma surgery (Figure 3). Their regenerative, immunomodulatory, and anti-inflammatory properties offer new hopes for managing OA, bone healing, and muscle-tendon injuries. Nevertheless, significant challenges related to EV characterization, delivery, dosing, and regulatory standardization must be addressed before widespread clinical application becomes viable.

### 8.1. EVs in Intervertebral Disc Degeneration

Intervertebral disc degeneration (IDD) is a major disease that causes chronic low back pain and other intervertebral diseases, such as herniation and stenosis, which create a societal burden worldwide [166]. The intervertebral disc (IVD) consists of three main parts: the inner gel-like nucleus pulposus (NP), surrounded by a fibrous ring annulus fibrosus (AF) and cartilaginous endplates (CEPs), which cover the IVD and ensure nutrient transport. IDD is a multifactorial process involving cellular senescence, inflammation, apoptosis, and ECM degradation [167]. Current treatments, including physiotherapy and surgical approaches, are directed toward symptoms and pain relief and have a temporary effect [168].

#### 8.1.1. EVs from Different Sources for IDD Repair

EVs have shown potential as a therapeutic approach for targeting IDD. EVs can repair IDD by influencing various mechanisms involved in molecular and cellular processes. This involves the suppression of inflammation, oxidative stress, and cell death, as well as the promotion of ECM production and stimulation of cell growth [169,170].

Currently, MSC-derived EVs are the most studied for IDD applications. The sources of MSC-EVs include bone marrow MSCs (BMMSCs), adipose-derived MSCs (AMSCs), umbilical cord-derived MSCs (UCMSCs), and others [171]. BMMSCs are the most widely distributed source of EVs. Several studies have demonstrated the therapeutic effects of EVs in in vivo IDD models. BMMSC-EVs reduced IDD progression, ECM degradation, delayed senescence, and improved disc height [172,173,174]. Furthermore, the preconditioning of BMMSCs under low oxygen conditions was demonstrated to affect the released EVs and their cargo. EVs derived from BMMSCs cultured under 2% oxygen conditions upregulated apoptosis and autophagy-related Bcl-2 interacting protein 3 (BNIP3) levels in NP cells compared to normoxic (21% oxygen) conditions. This shows that preconditioning of BMMSCs under hypoxic conditions improves the regenerative properties of EV cargo [174]. AMSC-derived EVs also showed great potential for IDD in vivo. These EVs diminished the senescence of IVD cells and ameliorated the progression of IDD in an established model [175]. UCMSC-EVs were shown to improve the histopathological structure of IVD in a rat tail IDD model. Additionally, UCMSC-EVs increased the expression of COL2A and mitochondrial transcription factor A (TFAM), showing their therapeutic ability through the modulation of mitochondrial dysfunction and oxidative stress of human NP cells in vitro [176].

IVD cells could also serve as a potential EV source. Culturing NP cells under different conditions could enhance the regenerative potential of EVs. NP cells cultured under Tie2-enhancing conditions demonstrated higher regenerative potential compared to those from standard NP cell cultures. Furthermore, this type of EV significantly overcame the effect of BMMSC-derived EVs in a coccygeal IDD rat model [177]. CEP MSCs (CEPMCs) have very similar properties compared to BMMSCs. CEPMSC-EVs play an important role in maintaining homeostasis between CEP and NP cells. Furthermore, CEPMSC-EVs from young individuals showed a better effect when applied to rat tail IDD than senescent CEPMSC-EVs. In contrast, EVs from senescent CEPMSCs even worsened the IDD through the delivery of miR-29b-3p [178]. These findings demonstrate the importance of the source of EVs and how degenerative processes could impact the behavior of other tissues.

Another source of studied EVs is platelet-rich plasma (PRP). Both PRP and PRP-derived EVs (PRP-EVs) could be applied for the treatment of IDD; however, the mechanisms of action of PRP-EVs are less studied compared to other sources of EVs. Compared with PRP alone, PRP-EVs were more effective in treating IDD [179,180]. Currently, there is one clinical trial in which EVs from blood are tested for IDD as intradiscal injections to NP, combined with PRP (NCT04849429) [181]. However, the results have not yet been published. Table 2 represents the application of different source EVs for IDD.

While EVs show promise, intradiscal injections may face limitations, such as the rapid clearance of EVs from the injection site and the necessity for repeated injections [185]. To overcome these limitations, biomaterial-based delivery systems, such as injectable hydrogels, are being developed to enhance EV retention and provide controlled release, improving their therapeutic efficacy [182,186]. It was shown that EVs embedded to thermosensitive hydrogel had a slower degradation rate compared to EVs without hydrogel [182]. Furthermore, the combination of EVs with a functional matrix hydrogel containing an arginine-glycine-aspartic acid (RGD)-complexed and decellularized NP matrix prolonged EV retention in vitro and ex vivo. Injections of conjugated EVs with this hydrogel into a rat IDD model maintained IVD height and reduced matrix changes [187].

#### 8.1.2. EVs as Delivery System

Although EVs offer numerous advantages and represent a promising cell-free therapeutic approach, they can also be employed as carriers for targeted drug delivery or bioactive molecules that modulate IDD. This expands their therapeutic potential and enhances the opportunities for personalized applications.

EVs can be designed to carry different biomolecules that target specific mechanisms of IDD. Transmembrane protein vasorin-containing MSC-derived EVs were shown to promote the proliferation of human primary NP cells. Additionally, they increased the expression of ACAN and COL2A1, while decreasing MMP3 and MMP13 through the Notch1 signaling pathway [182]. Genetically engineered EVs containing chondrocyte-affinity peptides and carrying the antioxidant transcription factor Nrf2 had a promising effect on CEP degeneration in a rat model. Affinity peptides enhanced the targeted affinity of EVs for CEP. Furthermore, modified EVs reduced CEP degeneration and IDD progression, evidenced by decreased expression of cleaved caspase-3 and BMP-2 [188]. The delivery of thioredoxin through MSC-derived EVs also showed promising results for IDD. BMMSC-derived EVs contain thioredoxin, which is important for the regulation of cellular senescence. In an in vivo rat IDD model, the application of these EVs maintained water content in NP and reduced degenerative histological scoring and maintained disc height [189].

In most cases, EVs are designed to deliver specific miRNAs to IDD. Many miRNAs were downregulated in IDD compared to healthy IVD. Targeting this issue with the delivery of specific miRNA-containing EVs could serve as a targeted therapeutic approach. The effect of specific miRNA-containing EVs in in vivo models is presented in Table 3.

Circular RNA circ_0072464 was found to be downregulated in IDD. These RNA-delivering BMMSC-derived EVs reduced IVD lesions in an IDD mouse model, reduced MMP13 levels, and increased collagen II levels [197]. Despite carrying specific IDD-associated miRNA, EVs can also carry miRNA inhibitors. For example, while miR-4450 was upregulated during IDD, the delivery of antagomiR-4450 by EVs reduced its expression, subsequently deceasing inflammation, inhibiting apoptosis, and promoting ECM restoration [198].

#### 8.1.3. Limitations and Challenges

**Figure 4:**
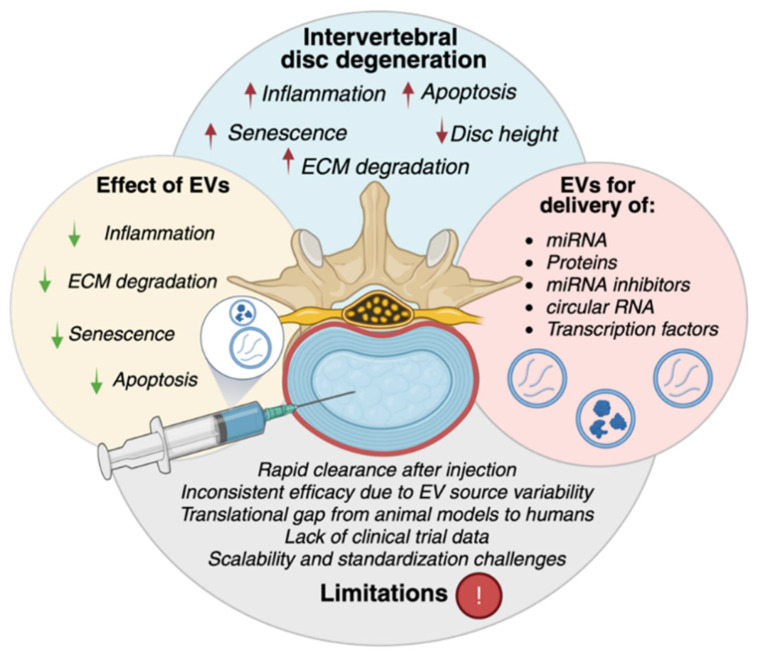
Main findings and limitations of the potential clinical application of EVs for IDD (created with Biorender.com).

While EVs show promising effects on IDD, there is a notable absence of clinical trials evaluating the safety and efficacy of these applications [199]. Currently, there is no FDA-approved treatment for IDD based on EVs [200]. The use of EVs at the clinical scale presents additional challenges: their composition and therapeutic efficacy can vary significantly depending on the cellular source, potentially leading to inconsistent outcomes. Moreover, scalability for clinical use differ from in vivo to animal studies, posing further translational hurdles [200]. Most preclinical in vivo studies are performed in small-scale animals, particularly rat tail IDD models, which have notable limitations in translating to human IVD conditions. Additionally, the NP in rodents retains more notochordal cells than that in humans, leading to a relatively slower degeneration rate [201]. Applying EVs to human IDD presents additional challenges as mechanical forces and intradiscal pressure can lead to the rapid clearance of EVs from the injection site [185]. Furthermore, the selection of a delivery vehicle, such as hydrogel, is critical for retaining EV bioactivity and enabling sustained release. Therefore, overcoming these translational challenges through optimized delivery systems, standardized EV production protocols, and robust large-animal or human-relevant models is crucial for advancing EV-based therapies for clinical application in IDD. Figure 4 summarizes the main findings and limitations of the clinical application of EVs for IDD. From a translational standpoint, clinically meaningful outcomes in musculoskeletal EV-based interventions typically include functional recovery (e.g., range of motion and load-bearing capacity), pain reduction, and imaging- or histology-based evidence of tissue regeneration and structural integrity. Delivery route represents an additional key determinant of efficacy, with most preclinical studies relying on local administration (e.g., intra-articular or intra-lesional injection) to maximize target engagement while limiting systemic exposure. Importantly, safety monitoring should include the assessment of local inflammatory reactions, ectopic tissue formation, immune responses, and potential off-target effects. Future clinical studies will require standardized outcome reporting and longer follow-up to better define the efficacy and safety profiles across different orthopedic indications.

## 9. EVs in Stroke Treatment

### 9.1. Exosomes in Ischemic Stroke

Exosomes are capable of crossing the BBB and can deliver proteins, mRNAs, and miRNAs to cells of the neurovascular unit (NVU), thereby contributing to the maintenance of central nervous system (CNS) homeostasis and enabling crosstalk between the brain and peripheral organs through circulating body fluids [202]. In preclinical models of stroke, exosomes derived from diverse cellular sources, including mesenchymal stem cells, neural progenitors, and endothelial cells, have been shown to promote tissue repair, attenuate apoptosis and inflammation, and preserve both neuronal and vascular integrity [202]. Beyond their regenerative potential, exosomes are also being investigated as diagnostic and prognostic biomarkers of stroke, given that their cargo reflects dynamic changes in the ischemic brain [203].

Exosomes can be engineered to enhance their therapeutic efficacy in oxidative stress–related injury by loading them with antioxidant systems. One strategy involves a manganese–organic framework nanoenzyme modified with polydopamine (pdA-MNOF), which mimics the catalytic domain of endogenous superoxide dismutase (SOD) and upregulates endogenous antioxidant enzymes [204]. In preclinical studies, pdA-MNOF demonstrated potent anti-inflammatory and antioxidant activity, markedly reducing reactive oxygen species (ROS) accumulation and protecting cells from oxidative damage [204]. Mechanistically, this effect is mediated through signal transducer and activator of transcription 3 (STAT3)-dependent upregulation of heme oxygenase-1 (HO-1) and superoxide dismutase 2 (SOD2), with downstream induction of vascular endothelial growth factor (VEGF), promoting angiogenesis [204]. In vivo, treatment with pdA-MNOF significantly improved outcomes in a middle cerebral artery occlusion (MCAO) model in mice by reducing lesion volume, increasing survival, and facilitating neurological recovery.

In parallel, extracellular vesicles derived from the gut probiotic *Lactobacillus reuteri* (LrEVs) have shown promise in stroke therapy by sensing elevated ROS levels and modulating the immune microenvironment through gut–brain axis signaling, including vagal nerve-mediated anti-inflammatory effects [205]. miRNAs can provide neuroprotection and promote repair following stroke. Several exosomal miRNAs—including miR-369-3p, miR-493-3p, miR-375-5p, and miR-1296-5p—have emerged as promising biomarkers for large-artery atherosclerotic stroke [203]. When assessed in combination, these miRNAs provide higher diagnostic accuracy compared to individual markers [203]. Notably, upregulation of exosomal miR-493-3p and miR-1296-5p has been correlated with lower NIH Stroke Scale (NIHSS) scores, suggesting a potential link between circulating exosomal miRNA profiles and stroke severity [203].

The inhibition of cell death represents a critical component of stroke therapy. Relevant to this goal, exosomes derived from healthy serum have been shown to confer neuroprotection in experimental stroke models, in part by suppressing endothelial apoptosis and preserving BBB integrity through autophagy-dependent mechanisms [206]. Specifically, these serum-derived exosomes inhibit endothelial apoptosis by shifting the balance of B-cell lymphoma 2 (Bcl-2) to Bcl-2-associated X protein (Bax) in favor of cell survival and by preventing caspase-3 activation via promotion of Akt (protein kinase B) phosphorylation [206]. These processes collectively contribute to enhanced endothelial resilience and vascular stability in the ischemic brain.

Beyond their antiapoptotic effects, EVs also support endothelial survival by enhancing energy metabolism and mitochondrial function. EV-mediated transfer of intrinsic mitochondrial components, such as mitochondria or mitochondrial DNA (mtDNA), has been shown to elevate intracellular ATP levels in human cerebral microvascular endothelial D3 (hCMEC/D3) monolayers [207,208,209]. These findings suggest that EVs can act as carriers of mitochondrial content and regulators of metabolic homeostasis in recipient cells under ischemic stress.

In addition, infarct-preconditioned exosomes have demonstrated superior efficacy in stroke treatment compared to naïve exosomes [207]. These vesicles promote vascular remodeling and enhance neurological recovery following stroke [207]. Mechanistically, infarct-preconditioned exosomes confer greater resistance to oxygen-glucose deprivation/reoxygenation (OGD/R), reduced apoptosis, and improved migratory capacity via VEGF and CXCR4 upregulation, enhancing angiogenesis and cell recruitment to the ischemic site [207].

### 9.2. Exosomes in Hemorrhagic Stroke

Exosomes are increasingly recognized as promising therapeutic candidates in hemorrhagic stroke too, particularly in intracerebral hemorrhage (ICH). Preclinical studies using BMMSCs have shown that exosomal miRNAs can exert both neuroprotective and anti-inflammatory effects. For example, miR-21-enriched exosomes protect neurons by regulating transient receptor potential melastatin 7 (TRPM7) and the nuclear factor kappa B (NF-κB) signaling pathway [208]. Exosomes loaded with miR-133b enhance motor recovery by suppressing Ras homolog family member A (RhoA) and activating the extracellular signal-regulated kinase/cAMP response element-binding protein (ERK/CREB) pathway [209]. Similarly, exosomes enriched in miR-146a-5p attenuate the microglial proinflammatory phenotype and neuronal apoptosis by inhibiting interleukin-1 receptor-associated kinase 1 (IRAK1) and nuclear factor of activated T-cells 5 (NFAT5). Exosomes carrying miR-183-5p further suppress neuroinflammation through modulation of programmed cell death 4 (PDCD4) and NOD-, LRR-, and pyrin domain–containing protein 3 (NLRP3) inflammasome [210]. Beyond microRNA cargo, MSC-derived exosomes have been shown to promote angiogenesis, axonal sprouting, oligodendrocyte proliferation, and remyelination, collectively supporting structural and functional recovery after ICH [211,212,213]. In addition, exosomes from human umbilical cord MSCs (hUC-MSCs) inhibit apoptosis and enhance cell proliferation, although evidence remains limited to small-animal models [214,215].

MSCs and their EVs have also been investigated in subarachnoid hemorrhage (SAH), another major subtype of hemorrhagic stroke, although most mechanistic insights derive from ICH models. Robust preclinical evidence and early clinical safety data support their potential therapeutic use in hemorrhagic stroke more broadly [216,217]. Through paracrine signaling, MSCs secrete cytokines, growth factors, and EVs that reduce proinflammatory cytokines such as interleukin-6 (IL-6) and interferon-γ (IFN-γ), suppress immune cell activation, and enhance neuronal migration and synaptogenesis. In IHC models, hepatocyte growth factor (HGF)-overexpressing hUC-MSCs were shown to promote remyelination [214], while MSCs engineered with glial cell line-derived neurotrophic factor (GDNF) increase synaptic proteins such as post-synaptic density protein-95 (PSD-95) and synaptophysin, thereby improving neurological recovery [218,219]. Similarly, BMMSCs reduced tissue loss and enhanced synaptogenesis in ICH rodents [219]. Evidence specific to SAH is more limited but indicates that MSCs mitigate early brain injury (EBI) by promoting an anti-inflammatory microglial phenotype and reducing neuronal apoptosis [220].

EVs can also contribute to secondary injury in hemorrhagic stroke. For instance, serum amyloid A1-containing EVs exacerbate neuroinflammation through TLR4/NF-κB activation [221]. MSC-derived EVs counteract such effects by modulating heat shock protein family A member 5 (HSPA5) and glutathione peroxidase 4 (GPX4) pathways to reduce ferroptosis [222]. Similarly, macrophage-derived EVs enriched in arginase-1 promote microglial phagocytosis and hematoma clearance, reducing lesion volume after intravenous administration in ICH models [223]. In SAH, pathogenic EVs contribute to vasospasm and EBI by impairing endothelial function, whereas MSC-EVs mitigate these effects through the delivery of miR-140-5p. This miRNA targets histone deacetylase 7 (HDAC7) to suppress microglial activation and neuronal apoptosis, leading to improved neurological outcomes in rodent studies [224]. Finally, EV-derived miRNA profiles also show diagnostic potential. In SAH, circulating and cerebrospinal fluid (CSF) levels of miR-9 have been associated with the development of vasospasm and delayed cerebral ischemia [225]. Similarly, miR-26b has been mechanistically linked to EBI and is being explored as a potential biomarker [226].

### 9.3. EVs and MSCs in Clinical Trials for Stroke

Several clinical trials have investigated EV- and MSC-based therapies in stroke, with most studies focused on safety, feasibility, and early signals of efficacy (Table 4). Although MSCs and EVs represent distinct therapeutic approaches, MSCs entered clinical testing earlier, and many of their therapeutic effects are believed to be mediated by released vesicles (e.g., exosomes), providing the biological and translational framework for current EV-based approaches.

Early-phase studies have demonstrated the feasibility and safety of MSC-based approaches for ischemic stroke, with studies showing promising functional or imaging benefits. The phase II randomized, double-blind trial NCT01678534 tested IV allogeneic adipose-derived MSCs in moderate-to-severe acute ischemic stroke (AIS), showing safety, feasibility, and preliminary neurological and radiological improvement. Similarly, NCT01297413 evaluated IV allogeneic bone marrow-derived MSCs in chronic ischemic stroke, reporting safety and modest but significant functional gains at 12 months. The ISIS-HERMES trial (NCT00875654) confirmed the safety and feasibility of IV autologous BMMSCs in subacute ischemic stroke (80% feasibility, no excess adverse events) with significant motor-specific improvements (Motor-NIHSS, Fugl–Meyer scores, and motor cortex fMRI activation), although no benefit was seen in global measures such as the NIHSS, Barthel Index, or functional outcome [227]. These findings suggest motor recovery may be driven by neuroplasticity.

The STARTING-2 trial (NCT01716481) tested autologous MSCs expanded with acute-phase serum in ischemic stroke patients with persistent deficits. The findings showed significantly greater motor recovery in the MSC group (higher Fugl–Meyer score improvement ratios), preservation of corticospinal tract integrity, and enhanced interhemispheric and ipsilesional connectivity, supporting a neurobiological mechanism of motor recovery [228]. In the phase II trial NCT01461720, IV autologous BM-MSCs in middle cerebral artery infarcts were safe and well tolerated, but no functional improvements were seen at 12 months, though a significant reduction in infarct volume change was observed [229].

In intracerebral hemorrhage, the Mayo Clinic phase I trial (NCT03371329) tested IV and intrathecal BMMSCs in supratentorial ICH, demonstrating safety and feasibility. The study confirmed IV BMMSC infusion was safe but highlighted the need for larger placebo-controlled studies [216].

In terms of clinical and biomarker outcomes, clear efficacy signals remain limited. The phase I/II trial NCT05292625 tested IV or intrathecal cord-derived MSCs in patients with post-stroke sequelae and, although safe, showed no efficacy and highlighted that tissue factor is not a reliable biomarker of MSC-induced hypercoagulation [230]. The phase I/II trial NCT03384433 investigated the stereotactic intracerebral administration of MSC-derived exosomes enriched with miR-124 in AIS; although completed, the results have not yet been published. Similarly, the observational NCT05370105 trial assessed circulating EV subpopulations as rehabilitation biomarkers, but the findings remain unknown. Other completed studies include NCT04063215, testing autologous adipose-derived MSC infusions (HB-adMSCs) in traumatic brain injury and hypoxic–ischemic encephalopathy (completed in 2024, results pending), and NCT02564328, a phase I trial of IV autologous BM-MSCs in chronic ischemic stroke, for which the results remain unreported.

Several ongoing studies are expected to provide further insights in the future. The ElViS-ACS trial (NCT06319742) and NCT05645081 are profiling circulating and endothelial-derived EVs, respectively, to distinguish ischemic stroke, TIA, and stroke mimics or to predict recurrence risk over 12-month follow-up. NCT06871800 (PRISMA) is evaluating blood EV profiles as predictors of recovery in rehabilitation after stroke or severe brain injury.

Many trials testing EV-based approaches to assess potential benefits in patients with stroke and related cerebrovascular conditions are still ongoing. The STEVIA trial (NCT06995625) is a phase I, open-label, dose-escalation study testing the safety and tolerability of the stem cell-derived EV therapy SNE-101 in AIS, with estimated completion in March 2027. Similarly, NCT05158101 is a phase I study of intranasal umbilical cord-derived MSC exosomes (AlloEx) in stroke patients, expected to be completed in February 2026. NCT07143786 is evaluating intravenously administered exosomes derived from induced neural stem cells (iNSC-EV01) within one week of ischemic stroke onset, with completion anticipated in mid-2027. The ExoCURE trial (NCT06138210) is a phase I randomized, double-blind, placebo-controlled study assessing the safety, tolerability, and preliminary efficacy of hiPSC-derived exosomes (GD-iExo-003) given intravenously within 1–7 days after AIS. Similarly, NCT06612710 is testing intravenously administered induced neural stem cell-derived exosomes (NouvSoma001) in AIS, with final completion expected November 2027.

Meanwhile, several MSC-based approaches remain active as well. NCT04434768 is a phase I trial of intravenous and intra-arterial umbilical cord-derived MSCs (UMSC01) in AIS, expected to finish in 2026. NCT05850208 is testing autologous BMMSC transplantation in ischemic stroke, while NCT04590118 (ASSIST) is assessing allogeneic MSC injections in chronic stroke. Other studies include NCT06862388 (umbilical cord MSCs in subacute ICH, not yet recruiting), NCT06518902 (repeated cord-derived MSC injections in AIS, not yet recruiting), NCT04093336 (single IV infusion of cord-derived MSCs in acute ischemic stroke), NCT06997939 (autologous BMMSC therapy for motor recovery after ischemic stroke, not yet recruiting), and NCT06129175 (Stroke Neuroncell-EX), a phase II/III double-blind trial testing IV allogeneic cord MSCs in AIS patients not eligible for reperfusion that is still recruiting. Finally, NCT02580019 is a phase II trial of repeated IV cord MSC infusion within three months of stroke onset, though its current status is listed as unknown.

Collectively, clinical studies of EV and MSC therapies in stroke remain in the early stages, with most completed trials establishing safety and feasibility but showing domain-specific benefits (e.g., motor recovery, neuroimaging biomarkers) rather than global functional improvements. Ongoing and upcoming phase I–II studies, particularly those testing exosome-based therapies and biomarker applications, are designed to refine safety, delivery, and patient selection. Large, placebo-controlled, multicenter trials will be critical to determine whether these biologics can achieve clinically meaningful recovery in stroke.

Nevertheless, these examples underscore the therapeutic promise of exosomes as natural nanocarriers for stroke treatment. Moving toward clinical translation, innovative approaches are being developed to optimize exosome-based therapies. One such strategy involves genetically infused, functionally tailored exosomes (GIFTed-Exos), which exhibit improved incorporation of membrane proteins and enhanced loading of soluble protein cargos. Alongside these engineered platforms, stem cell-derived exosomes, most notably those from MSCs, remain the most advanced candidates, supported by preclinical data and ongoing clinical exploration. Researchers are particularly drawn to exosomes because they combine the therapeutic breadth of cell therapies with the scalability, safety, and precision of acellular products. Their versatility—including the ability to cross the BBB, home to infarcted brain regions, and deliver bioactive molecules that exert neuroprotective and proregenerative effects— positions them as frontrunners for future translation into stroke therapies [231].

## 10. EVs in Autoimmune Diseases

The quantity, tissue origin, molecular composition, and functional properties of extracellular vesicles are closely linked to specific diseases and their progression, making EVs promising candidates for a wide range of biomedical applications, including diagnostics and therapeutics. By mediating intercellular communication, EVs mirror the physiological and pathological states of their cells or tissues of origin. They influence key processes such as immune responses, cell cycle regulation, autophagy, and oxidative stress. Increasingly recognized as potent immune modulators, EVs play a critical role in the development and perpetuation of autoimmune diseases [164,232].

Because EVs circulate in bodily fluids, exhibit remarkable stability, and carry disease-specific molecular cargo, they offer a non-invasive window into cellular health and immune dysregulation. In autoimmune disorders, where the immune system mistakenly targets self-tissues, EVs can exacerbate disease by transporting autoantigens that activate autoreactive T- and B-cells [164,233]. The role of EVs has been documented in multiple autoimmune diseases (Table 5).

Another reason EVs are promising as diagnostic biomarkers, including for autoimmune disorders, is their increased release under pathological conditions (Table 6). Because their cargo is dynamically reprogrammed according to the secreting cell’s state, EVs can track disease onset, progression, and therapeutic responses [232,234].

Beyond biomarker discovery, EVs are being actively explored as therapeutic agents in autoimmune diseases. Their cargo can be reprogrammed based on the physiological state of the secreting cells, which allows them to reflect disease progression and treatment effects. Their low immunogenicity, intrinsic targeting ability, and ease of manipulation position them as attractive cell-free therapies. They can exert immunosuppressive or immunostimulatory effects, such as anti-inflammatory, antimicrobial, or antitumor effects, or as an alternative to mesenchymal stem cell transplantation. EVs can also function as nanocarriers for natural nanocarriers, delivering nucleic acids, proteins, and small molecules to specific target cells to target cells, enhancing existing therapies [234].

For example, in RA, EVs have been engineered to deliver anti-inflammatory molecules (e.g., miR-100-5p, IL-10, IL-4) to reduce inflammation and inhibit RA-related cell proliferation. EVs loaded with curcumin modulate inflammatory responses in RA fibroblast-like synoviocytes (RA-FLSs). In RA, EVs can also be used to modulate the local microenvironment and promote tissue regeneration with their anti-inflammatory properties. MSC-EVs carrying various miRNAs, lncRNAs, and circular RNAs (circRNAs) have been shown to suppress inflammation and the pathogenic activities of FLSs. For example, EVs from human umbilical cord MSCs containing miR-451a can inhibit the proliferation and migration of RA synovial fibroblasts, improving arthritis in rat models [235].

IBD therapy is another example of EVs as a therapeutic agent, whether for targeted immunomodulation, tissue repair and regeneration, or advanced drug delivery. EVs promote a shift from proinflammatory M1 macrophages to anti-inflammatory M2 phenotypes via delivery of miR-216a-5p, suppressing HMGB1/TLR4/NF-κB signaling. EVs also modulate T-cell differentiation toward regulatory phenotypes. EVs possess regenerative capabilities by delivering their cargo to damaged intestinal tissue. This promotes healing, reduces colonic fibrosis, and restores the integrity of the mucosal barrier. For example, EVs containing miR-200b have been shown to prevent epithelial-to-mesenchymal transition (EMT) by downregulating fibrosis-related proteins ZEB1 and ZEB2, which is critical in preventing complications like stricture formation in Crohn’s disease. EVs are highlighted as a superior platform for drug delivery compared to synthetic nanoparticles. In the context of drug delivery, EVs can be loaded with therapeutic molecules like anti-inflammatory drugs or siRNAs targeting TNF-α, delivering them precisely to the site of inflammation to enhance efficacy and reduce systemic toxicity. This is possible due to EVs’ natural origin and inherent targeting capabilities, which minimize off-target effects and immunogenicity [236].

Undoubtedly, extracellular vesicles offer significant promise as non-invasive biomarkers and as innovative therapeutic agents. Nevertheless, their clinical translation is currently hindered by challenges, including heterogeneity of EV populations, incomplete understanding of their molecular mechanisms, and the absence of standardized isolation and characterization protocols. Addressing these gaps through large-scale validation studies, advanced omics approaches, and the development of robust multiplexed diagnostic assays will be essential to fully unlock the potential of EV-based applications. With continued innovation and interdisciplinary collaboration, EV-based biomarkers and therapeutics could redefine how autoimmune diseases are diagnosed, monitored, and treated, ushering in a new era of personalized and targeted care.

## 11. EVs in Chronic Wound Patients

Chronic wounds are a result of the inability to restore structural and functional skin integrity due to lack of progression through timely and orderly phases of wound repair [237]. Proper diagnosis and treatment of these wounds are imperative, as chronic wounds represent a global health problem that accounts for a diminished quality of life and significant healthcare costs [238,239]. Examples of chronic wounds include pressure ulcers, venous leg ulcers, diabetic foot ulcers, and arterial ulcers [239,240]. While these wounds are more commonly associated with underlying comorbidities, chronic wounds can also result from localized injuries due to burns, trauma, infection, and radiation [241,242].

The mechanisms underlying chronic wounds include persistent inflammation and dysfunctional cellular responses. Sustained activation of cytokines and matrix metalloproteinases promotes structural changes in endothelial structures and extracellular matrix degradation, thereby impairing angiogenesis [243]. Microvascular dysfunction and tissue hypoxia can also predispose patients to chronic ulcers due to pathologically altered vein wall changes and vein distension [243]. Furthermore, elevated reactive oxygen species levels can contribute to additional tissue necrosis and oxidative stress, which is exemplified in chronic burn wounds [244].

Sensory and autonomic neuropathies in diabetic patients contribute to the development of chronic diabetic ulcers. Sensory neuropathy can lead to skin breakdown through loss of protective sensation, while autonomic neuropathy may result in vasoelastic changes [245]. Systemic risk factors that can contribute to non-healing wounds include vascular insufficiency, advanced age, compromised nutritional status, immunosuppression, and chronic mechanical stress [237].

MSCs have been considered crucial for neovessel formation and share features of pericytes important in stabilizing the vasculature across various tissues [246]. Both MSCs and pericytes express CD146^+^, which has identified a subpopulation that provides hematopoietic support and exhibits a preferential perivascular topography [246,247]. The effects of MSCs in neovascularization and angiogenesis are largely mediated by their paracrine actions. This includes the secretion of key factors like vascular endothelial growth factor (VEGF) and MMP-1, which have been shown to induce capillary-like structure formation in hypoxic environments [247].

Mesenchymal stem cell extracellular vesicles (MSC-EVs) offer a potential therapeutic tool for promoting the healing of chronic wounds by modulating inflammatory responses, promoting re-epithelization, inducing angiogenesis, and mediating cell-to-cell communication [242,248]. However, some limitations persist, including a lack of standardized protocols for MSC-EV isolation and an absence of standardized manufacturing methods [249]. Another limitation is MSC-EV heterogeneity, as MSC-EVs derived from various cell types, or even from the same cell type, can exhibit variable therapeutic functions [248,249].

### 11.1. Mechanisms of Increased Wound Healing with MSC-EVs

To understand the applications of MSC-EVs in chronic wounds, it is important to investigate the different mechanisms by which they can promote wound repair. One mechanism by which MSC-EVs play a role in wound repair is through the promotion of angiogenesis. Typically, angiogenesis is activated to stimulate the proliferation of endothelial cells in response to local stressors such as hypoxia and acidity [250]. The formation of new vessels alleviates the lack of oxygenation and reduces lactic acid buildup. MSC-EVs contain many components of cell signaling, including proteins, DNA, and RNA, which interact with processes such as angiogenesis. Angiogenesis occurs under a specific balance of pro- and antiangiogenic factors [251]. The proangiogenic mediator vascular endothelial growth factor (VEGF) is found within MSC-EVs and has widespread effects on wound repair including the promotion of angiogenesis, epithelization, and collagen deposition [252]. In a full-thickness wound model in rats, it was found that upregulation of VEGF165 expression resulted in greater wound area reduction and collagen deposition [253].

However, VEGF levels are dysregulated in chronic wounds, where there is heightened proteolytic activity and degradation of VEGF as well as inhibition by VEGFR-1 [254]. In chronic wound patients, VEGF is limited in its potential to promote angiogenesis and wound repair due to the inflammatory environment. In a systematic review that investigated the stimulation of VEGF in patients with chronic diabetic foot ulcers, it was found that VEGF administration resulted in an increased rate of wound healing [255]. As VEGF has a short half-life between 4 and 24 h, the review discussed potential means of delivery in the form of gels or coatings to enable longer-lasting effects [255]. Notably, MSC-EVs contain a lipid bilayer that serves to protect cargo from enzymatic degradation [256]. Given there is excessive enzymatic degradation in chronic wounds, MSC-EVs could be an essential method in transporting and protecting the EVs to enable wound healing effects.

Perhaps an even more critical pathway by which MSC-EVs can impact healing in chronic wounds is by their immunomodulation effects. MSC-EVs influence the extent of inflammation via macrophage polarization in the microenvironment of a chronic wound and reduce its proinflammatory state [257]. One study found that exposure to MSC-EVs increased the polarization ratio of M2 macrophages, the anti-inflammatory phenotype, compared to the M1 proinflammatory phenotype [257,258]. The mechanism driving the anti-inflammatory polarization of macrophages in diabetic mice was the MSC-EV activation of the PTEN/Akt signaling pathway and the downregulation of proinflammatory cytokine gene expression (IL-1β, TNF-α, and iNOS). MSC-EV cargo also was shown to upregulate the expression of IL-10 and Arg-1 in diabetic mice, which promoted the polarization of M2 macrophages [258]. The anti-inflammatory M2 macrophages release cytokines that modulate the production of regulatory T-cells that can suppress inflammation and catabolism [257]. Overall, MSC-EVs promote an anti-inflammatory environment in wounds through the proliferation of M2 macrophages and inhibition of proinflammatory cytokines and M1 macrophages. In chronic wounds where immunomodulation is disrupted via unbalanced cytokines and growth factors that favor a proinflammatory environment, MSC-EVs are promising agents that can be manipulated to promote wound repair.

MSC-EVs also promote wound repair through fibroblast activation. In an experimental study that investigated MSC-EVs in vitro, increased fibroblast proliferation was observed [259]. The MSC-EVs were found to contain STAT3, an integral protein involved in signaling pathways for angiogenesis, cell proliferation, and migration [259]. Fibroblast exposure to MSC-EVs resulted in the activation of additional pathways involved in cell proliferation and wound repair such as AKT and ERK ½ [259]. There was also an upregulation of the expression of growth factor genes and the production of HGF, IL-6, IGF1, NGF, and SDF1 with the use of MSC-EVs [259]. Despite the limited repair pathways initially present in the fibroblasts of chronic wound patients, there was dose-dependent fibroblast activation and migration seen with the administration of MSC-EVs [259].

Additionally, it has been demonstrated that MSC-EVs promote antiscarring effects. In one in vitro (human fibroblasts) and in vivo model (mouse), adipose-derived MSC-EVs resulted in decreased scar length and thickness [260]. Similarly, another study found a reduction in scar width of mouse wounds with the application of human umbilical cord-derived MSC-EVs [261]. Additionally, there was an increased type III to type I collagen ratio in mouse skin tissue exposed to MSC-EVs [260]. In parallel, in a different study, there was an increased ratio of type III collagen mRNA to type I collagen mRNA [261]. There was also greater ratio of TGF-β3 to TGF-β1 in vivo amongst the MSC-EV-exposed mice [260]. A supporting study found an increased expression of TGF-β3 mRNA to TGF-β1mRNA both in vivo and in vitro amongst the MSC-EV-exposed groups [261]. The increased ratios of type III collagen and TGF-β3 are both distinctive of scar-free tissues and reflect a fine reticular collagen pattern with reduced cross-linking [260,261,262].

### 11.2. Current Studies on MSC-EV Effects on Wound Healing

There is growing evidence demonstrating that MSC-EVs promote the healing of diabetic wounds via promotion of angiogenesis and suppression of the inflammatory response. One recent study showed that MSC-EVs decreased LPS-induced macrophage inflammation [263]. It was also shown that the combination of MSC-EVs with amino acid peptides increased the anti-inflammatory effect and, in addition, led to increased fibroblast proliferation and improved wound healing in diabetic rat wounds. Another study found that the injection of placenta-derived MSC-EVs into full-thickness diabetic mice wounds resulted in increased wound closure rates and decreased scar width [264]. Additionally, it was found that hyperglycemia-induced human dermal fibroblasts treated with MSC-EVs had increased proliferation, increased migration, and decreased apoptosis, which are all beneficial to the wound healing process [264].

Among MSC-EVs currently being investigated in diabetic cutaneous wounds, recent studies have primarily examined RNA types such as long non-coding RNAs (lncRNA) and micro-RNAs. For example, one study found that adipose MSC-derived lncRNA H19 influenced the polarization of macrophages from the M1 proinflammatory to the M2 anti-inflammatory type [265]. This is a necessary transition from the inflammatory to the proliferative stage of wound healing and is also a stage that is dysfunctional in diabetic wound healing [266]. Another study found that connective tissue growth factor (CTGF) was under-expressed in diabetic foot ulcer tissue in rats, and that lncRNA H19 increased the expression of CTGF, leading to increased angiogenesis and accelerated wound healing in addition to decreased apoptosis [267]. A diabetic mouse model showed that the injection of MSC-EVs overexpressing lncRNA H19 to diabetic cutaneous wounds promoted HaCaT cell line proliferation and migration and suppressed pyroptosis both in vitro and in vivo [268]. A study found that lncRNA KLF3-AS1-containing MSC-EVs stimulated proliferation and migration and inhibited apoptosis in human umbilical vein endothelial cells (HUVECs) that were challenged by high glucose conditions. In this in vivo diabetic mouse model, EVs from KLF3-AS1-expressing MSCs promoted wound healing as evidenced by increased angiogenesis, reduced inflammation, as well as decreased miR-383 expression, and upregulated VEGFA [269].

Alternatively, there are some micro-RNAs that are upregulated in diabetic wounds, and research has demonstrated that the inhibition of these micro-RNAs can be beneficial to wound healing. For example, recent evidence has demonstrated an upregulation of miR-155 in diabetic foot ulcers, which has been negatively associated with the rate of wound healing [270]. Numerous studies have also demonstrated that inhibiting the expression of miR-155 had a positive effect on wound healing in diabetic foot ulcers [271,272,273]. Gondaliya et al. loaded MSC-EVs with a miR-155 inhibitor using an in vitro human keratinocyte/dermal fibroblast model as well as an in vivo diabetic wound mouse model and found a decrease in inflammatory markers and MMPs in human keratinocyte cells with miR-155 inhibition [272]. Furthermore, there was increased angiogenesis, re-epithelialization, and wound healing in mice treated with miR-155-inhibited MSC-EVs. Another study found that the inhibition of miR-155 resulted in the increased expression of fibroblast growth factor 7 in the diabetic wounds of mice, as well as the acceleration of wound closure and re-epithelization [274]. This evidence suggests that increased levels of miR-155 in diabetic wounds has a negative effect on wound healing, and further, that inhibition of miR-155 expression may have beneficial effects on the healing of diabetic foot ulcers.

More recently, there has been emerging evidence on the use of MSC-EVs in the healing of more complex wounds, such as methicillin-resistant *Staphylococcus aureus* (MRSA)-infected wounds. In a 2025 study, MSC-EVs were embedded in a hydrogel with a self-adaptive release of EVs at wound sites based on pH and reactive oxygen species presence [275]. The use of this methodology resulted in a significantly increased wound closure rate, increased deposition of type I and type III collagen, and a more orderly arrangement of collagen fibers and fibroblasts compared to controls. This methodology led to increased wound closure and healing compared to EVs alone, which was attributed to the hydrogel’s additional antibacterial activity as well as the controlled release of EVs to the site of the wound [276]. In another model of mice with sclerodermatous chronic graft vs. host disease, it was found that MSC-EVs resulted in decreased pathologic fibrosis and macrophage activation.

While there are a handful of clinical trials investigating the use of MSC-EVs in chronic wounds, most previous work has been performed in animal models. Collectively, these studies have shown that MSC-EVs increase healing in various chronic wound types including diabetic ulcers and complex infected wounds. However, further investigation is needed to understand the differences in the expression of MSC-EVs in human chronic wound patients and to determine if the wound healing benefits of MSC-EVs translate to clinical studies.

## 12. EVs and Chronic Pain

Chronic pain is now formally recognized in the International Classification of Diseases (ICD-11) as a distinct disease entity, and it is subdivided into several categories based on the underlying pathophysiological mechanisms [277]. It affects hundreds of millions of individuals worldwide, imposing a substantial societal and economic burden [278]. Current pharmacological options offer only limited benefit for both chronic inflammatory and neuropathic pain and are frequently associated with significant adverse effects. For example, low back pain alone affects more than 600 million people globally and accounts for an estimated USD 50 billion in annual economic costs [279].

Against this backdrop, regenerative medicine and almost all exosome-based therapeutics have emerged as a promising disease-modifying strategy. Exosomes can target upstream mechanisms of chronic inflammatory pain and modulate neuropathic pain via novel biological pathways, while maintaining an excellent safety profile [280]. Moreover, several preclinical studies have shown that chronic pain states are associated with alterations in long non-coding RNAs (lncRNAs) and with distinct exosomal signatures, suggesting that these molecules may also serve as biomarkers for differentiating chronic pain phenotypes [281].

From a mechanistic standpoint, one of the most compelling applications of exosomes is their ability to deliver nerve growth factor (NGF) in a targeted fashion. Although NGF has well-established analgesic properties in neuropathic pain models, its systemic administration is limited by substantial adverse effects [282]. Recent work by Li et al. demonstrated in a chronic constriction injury (CCI) rat model that spinal administration of NGF-loaded exosomes (Exo-NGF) significantly attenuates tactile allodynia and thermal hyperalgesia while markedly reducing IL-18, IL-1β, and TNF-α expression [283]. These results suggest a mechanism-specific analgesic effect that is not attainable with current pharmacological treatments. Comparable findings—improvements in mechanical allodynia, thermal hyperalgesia, and reductions in proinflammatory cytokine release—have been reported using exosomes derived from mesenchymal stem cells (MSCs) and glial cell line-derived neurotrophic factor (GDNF) in the same animal model [284].

Strong preclinical evidence also indicates that exosomes, owing to their low immunogenicity and innate ability to cross the blood–brain barrier, represent ideal vectors for delivering therapeutic miRNAs capable of modulating spinal nociceptive processing while minimizing off-target toxicity [285].

Exosome-based approaches have also been shown to be promising in the treatment of low back pain. A recent meta-analysis evaluating exosome-loaded hydrogels in ten animal studies reported increases in intervertebral disc height at 4 and 8 weeks, enhanced type II collagen deposition, and simultaneous reductions in cellular senescence markers (p16Ink4a-positive and p21CIP1A-positive cells), highlighting their regenerative potential in disc degeneration [286].

Despite encouraging preclinical data—and some ongoing clinical trials in osteoarthritis that are discussed in other chapters of this article—controlled clinical studies in chronic pain are still lacking [287,288]. To date, only two uncontrolled clinical studies have been published [289,290]. In 2021, Philips et al. reported the peridural administration of bone marrow-derived MSC extracellular vesicles in five patients with cervical radicular pain, observing a 55% reduction in Brief Pain Inventory scores at one month [289]. Two patients experienced transient post-procedural headache and local pain, which resolved spontaneously. However, safety data for peridural delivery of such products remains insufficient, and the study was neither controlled nor registered in international trial databases.

A more informative contribution comes from a recent pilot study by Wilson et al., in which twenty patients with chronic low back pain received lumbar facet joint injections of an advanced investigational product consisting of bone marrow-derived MSC extracellular vesicles [290]. Across follow-up visits at 1, 3, 7, 14, 30, 60, and 90 days, the investigators reported an excellent safety profile (no adverse events) and clinically meaningful improvements, including statistically significant reductions in pain scores, a 65.04% improvement in the Severity Index, and a 58.43% improvement in the Oswestry Disability Index.

In summary, although high-quality clinical evidence is still absent, the rapidly expanding preclinical and animal data literature highlights the therapeutic potential of exosome-based interventions for chronic pain, particularly neuropathic pain. As the field advances, it will be critical that future trials adhere to established practice guidelines for regenerative medicine to ensure methodological rigor, safety, and reproducibility [291].

## 13. EVs in Dermatology

Exosome therapy represents an innovative approach in the field of hair regeneration including the treatment of alopecia. Current research has shown that novel therapy options can induce hair regrowth and regeneration, which can be shown clinically in increased hair thickness and density [292]. Moreover, mesenchymal and adipose stem cell-derived exosomes were shown to be very promising as regards hair regeneration, containing cytokines and growth factors that promote hair growth [293]. For example, the activation of WNT receptors (primarily Frizzled) located on the exosome surface can induce β-catenin activation, a crucial signaling pathway involved in hair morphogenesis and in the process of hair regeneration, thus promoting the anagen phase of the hair cycle and differentiation of keratinocytes [294]. Other studies are focusing on the research of exosomes derived from dermal papilla cells (DPCs), a mesenchymal type of adult stem cell that is involved in the regulation of hair follicle development and the hair growth cycle while interacting also with epithelial cells [295].

The therapeutic application of exosomes in dermatology, aesthetic medicine, and hair regeneration remains challenging. Standardization is needed across the entire production process, including the selection of exosome sources and manufacturing protocols (isolation, purification, characterization, and storage), as well as the determination of clinical indications and safety profiles [296]. Current studies have explored the use of exosomes for topical application, either alone or in combination with procedures such as microneedling or laser treatments [293]. Human blood- or tissue-derived exosomes have also been investigated for therapeutic purposes, including potential applications in hair loss management and broader dermatological conditions. Exosomes possess a complex composition of proteins, lipids, and growth factors that can support skin repair, hydration, and protection [297]. Additionally, exosomes and extracellular vesicles have demonstrated effects on skin aging, inflammatory conditions (such as psoriasis, atopic dermatitis, or acne), melanogenesis, and hyperpigmentation, as well as wound healing and scar formation [297].

### Safety and Adverse Events

Exosome-based therapies in dermatology, including hair restoration and skin treatments, are generally well tolerated, with reported adverse events being mild and mostly local, such as transient erythema, swelling, or pruritus at the application site [293,296]. No serious systemic adverse effects have been consistently reported in clinical studies of autologous or allogeneic exosomes [292,296]. Preliminary evidence suggests that exosomes derived from dermal papilla cells or platelet-rich plasma are safe when administered topically or via intradermal injection, though the heterogeneity of exosome sources, preparation methods, and dosing protocols limits broad conclusions about long-term safety [294,295]. Standardized manufacturing processes, rigorous characterization, and controlled clinical trials are needed to ensure reproducibility and to monitor for rare or delayed adverse events, including potential immunogenicity or unintended off-target effects [297].

## 14. EVs in Cardiology

The roles of EVs in atherosclerosis, myocardial infarction (MI), and heart failure (HF) extend beyond experimental interest, positioning EVs as candidates for clinical diagnostics and therapeutics [298,299]. Circulating EVs reflect cellular activation, injury, or stress, providing a liquid biopsy of the cardiovascular system [300]. Their cargo—including proteins and microRNAs (miRNAs)—changes dynamically during disease and may complement established biochemical and imaging markers [298,301].

In acute coronary syndromes (ACS), circulating extracellular vesicles (EVs) are increased in number and display enhanced prothrombotic activity, with thrombin generation correlating with externalized phosphatidylserine, EV counts, and total EV membrane surface area [302]. Cardiac EV-associated miRNAs such as miR-1 and miR-133a increase rapidly after myocardial injury, demonstrating potential for ultra-early MI rule-in when combined with high-sensitivity troponins [301,303].

In chronic coronary disease, platelet- and monocyte-derived EVs predict cardiovascular events beyond classical risk factors, enabling improved individual risk stratification [304]. In heart failure, distinct EV signatures correlate with New York Heart Association class and clinical outcomes, supporting possible use in disease staging and therapy monitoring [298,305].

Widespread clinical adoption, however, remains hindered by the lack of standardized EV isolation, quantification, and characterization methods [306]. Harmonized protocols are a prerequisite for regulatory approval and broader clinical implementation. EVs show promise as dynamic treatment-response indicators. Following percutaneous coronary intervention (PCI), changes in platelet-derived EV levels reflect periprocedural myocardial injury and antiplatelet drug effectiveness [307].

In cardiac surgery, cardiopulmonary bypass induces characteristic shifts in EV populations mirroring inflammatory and endothelial activation. These signatures could enable perioperative risk prediction and individualized post-operative management [308]. Serial EV profiling may also inform treatment decisions in HF and guide the response to device therapies, although clinical validation is ongoing [305].

### EVs as Regenerative and Cardioprotective Therapeutics

EVs derived from mesenchymal stromal cells or cardiac progenitor cells exert cardioprotective effects in preclinical MI models. They enhance angiogenesis, limit apoptosis, and improve left ventricular remodeling [299,309,310]. These benefits resemble those of cell-based therapies while avoiding the risks associated with cellular engraftment, including arrhythmogenic potential and immunogenicity [311].

Furthermore, engineered EVs represent a novel class of targeted delivery systems capable of transporting therapeutic nucleic acids, proteins, and small molecules directly to the injured myocardium [312]. Although cardiovascular clinical trials remain early-stage, feasibility and safety data from oncology applications support the translational potential of EV-based drug delivery [103].

EV-based approaches are expected to enter cardiovascular medicine primarily through their integration into comprehensive biomarker panels, where they will complement and enhance established diagnostic strategies. As the evidence base grows and the underlying technologies advance, their application is likely to expand into the realm of precision therapeutic delivery, including targeted modulation of post-myocardial infarction remodeling, reduction in myocardial inflammation, and potential intervention in inherited forms of cardiomyopathy. For these developments to progress successfully, several key challenges must be resolved. These include the establishment of standardized methods for extracellular vesicle isolation and benchmarking, the implementation of rigorous validation across multiple clinical centers, and the development of clear regulatory pathways appropriate for advanced medicinal therapies. It will also be essential to demonstrate that approaches based on extracellular vesicles provide meaningful added value compared with existing biomarker systems [306]. Despite these obstacles, extracellular vesicles offer unique biological and translational advantages as both diagnostic biomarkers and therapeutic carriers, positioning them as potentially transformative tools in the future of cardiovascular care.

## 15. Safety Considerations and Potential Risks of EV-Based Therapies

Despite their therapeutic promise, EV-based interventions may also pose clinically relevant safety risks that require careful consideration. EVs can transfer biologically active proteins, nucleic acids, and lipids capable of modulating recipient-cell behavior, and in specific contexts may contribute to protumorigenic signaling, malignant transformation, or cancer progression, particularly when EVs originate from tumor-associated or stressed cells. In addition, the lipid-rich EV membrane may carry lipid peroxidation products and oxidative damage-associated molecules, which could theoretically propagate oxidative stress in recipient tissues. Therapeutic strategies using EV-loaded miRNAs also require strict dose optimization and biodistribution control, since excessive miRNA delivery or unintended accumulation in off-target tissues may lead to unwanted gene regulation and adverse effects. Finally, EV manufacturing workflows must mitigate contamination risks, including inadvertent co-isolation of bacteria- or virus-associated vesicles or microbial components, highlighting the importance of sterile handling, validated purification procedures, and rigorous quality control aligned with current EV standardization recommendations. Overall, these considerations underline the need for cautious interpretation of preclinical efficacy results and for comprehensive safety evaluation prior to clinical translation.

## 16. Conclusions

Exosomes have emerged as versatile nanocarriers with immense potential across diverse clinical domains, including diagnostics, prognostics, and targeted therapy. Their ability to encapsulate bioactive molecules, such as proteins, lipids, and nucleic acids, and to reflect the physiological state of their cell of origin positions them as powerful biomarkers for early disease detection and monitoring. Furthermore, the capacity of engineered exosomes to deliver therapeutic cargo with high specificity and minimal immunogenicity offers a promising avenue for the development of next-generation drug delivery systems. Despite these advances, several challenges must be addressed before exosome-based tools can be fully integrated into clinical practice. These include the need for standardized isolation and characterization protocols, scalable production methods, and robust regulatory frameworks. Continued interdisciplinary research and well-designed clinical trials will be essential to unlock the full translational potential of exosomes and establish them as a cornerstone of personalized medicine.

## Figures and Tables

**Table 1:** Therapeutic applications of extracellular vesicles in otorhinolaryngology (ORL): a concise summary.

Indication	EV Source	Model/Application	Main Effects	References
Sensorineural hearing loss (SNHL)	Mesenchymal stromal cell-derived extracellular vesicles (MSC-EVs) (bone marrow, umbilical cord); heat shock preconditioned MSC-EVs	Mice, cisplatin ototoxicity; first-in-human intracochlear application	↓ hair cell apoptosis and oxidative stress; ↑ spiral ganglion neuron survival; improved auditory thresholds; feasibility/safety in human inner ear; HSP70-enriched EVs attenuate cisplatin ototoxicity	[73,74,75,76,77]
Vocal fold scarring	MSC-EVs; epidermal stem cell EVs	Rabbit model; polyethylene glycol (PEG)-fibrin hydrogel	Lamina propria restoration; reversal of fibroblast-to-myofibroblast transition; antifibrotic effects; improved biomechanics; mechanistic support from epidermal EVs	[78,79]
Airway inflammation	Epithelial and immune cell EVs; MSC/adipose (ASC)-EVs; engineered EVs (aptamer-decorated)	Mouse models; intranasal delivery; in vitro human epithelial cells	Epithelial/immune EVs: miR-21/155 → type 2 helper T-cell (Th2) inflammation; therapeutic MSC/ASC-EVs: restore epithelial barrier, ↓ eosinophilia, ↑ regulatory T-cells (Tregs); aptamer engineering ↑ mucosal retention/efficacy; nasal EV signatures support endotyping	[80,81,82,83,84,85]
Head and neck squamous cell carcinoma (HNSCC)	Tumor-derived EVs; engineered exosomes (miR-34a, siRNA against LCP1)	In vitro, xenograft models	Tumor EVs drive invasion, angiogenesis, immune evasion; engineered EVs ↓ proliferation, migration, angiogenesis; proof-of-concept delivery	[86,87,88]
Nasopharyngeal carcinoma (NPC)	Tumor-EVs (EBV-LMP1, viral miRNAs); engineered EVs (antagomiRs vs. EBV miRNAs)	In vitro, xenograft models	↓ angiogenesis/invasion; blockade of EBV-miRNA signaling; reduced therapy resistance; LMP1/miR-BARTs as tumor-linked cargo	[89,90,91]
Surgical complications (PCF after laryngectomy)	MSC-EVs (conceptual/experimental)	Preclinical/experimental settings	↑ fibroblast proliferation; ↑ angiogenesis; ECM remodeling; potential adjunct to flaps/advanced dressings in irradiated fields	[92]
Clinical feasibility	Autologous adipose-MSC EVs	Clinical study NCT04270006 (periodontitis)	Local feasibility/safety; supports near-term translation of EV therapeutics	[93]

**Table 2:** Application of EVs from different sources on IDD.

Source of EVs	Application	Approach	Effect	Reference
**BMMSCs**	Injection of EVs embedded in a hydrogel to rat tail IVD	Ex vivo	Reduced the degenerative score of the IVD (histological evaluation).	[182]
	Injection to rat tail IDD model	In vivo	Reduced IDD progression and prevented the ferroptosis of NP cells via the p62/KEAP1/NRF2 signaling pathway.	[183]
	Injection to rat tail IDD model	In vivo	Delayed NP cell senescence and promoted ECM synthesis.	[174]
	Rat tail IDD model	In vivo	Ameliorated endoplasmic reticulum stress and apoptosis of NP cells, diminished the progression of IDD, and improved disc height.	[173]
	Rat IDD model	In vivo	Improved disc height index, reduced apoptosis and ECM degradation.	[172]
**UCMSCs**	Rat tail IDD model	In vivo	Delayed the progression of IDD.	[176]
**AMSCs**	Injection of EVs with thermosensitive dECM@exo hydrogel to rat tail IVD	In vivo	Regulated ECM synthesis and degradation by controlling MMPs and diminished inflammatory response.	[184]
	Rat IDD model	In vivo	Rejuvenated senescent NPC CEP cells and reduced IDD.	[175]
**Young NPCs**	Coccygeal IDD rat model	In vivo	Preserved disc height index, attenuated degenerative changes, and significantly reduced mechanical hypersensitivity.	[177]
**CEPMSCs from young patients**	Intradiscal injection to rat IDD tails	In vivo	Increased expression of ACAN and COL2 in NP tissue and maintained IVD height.	[178]
**Blood**	Intradiscal injection to NP with PRP	Clinical trial	Results not published yet.	[181]
**PRP**	Rat IDD	In vivo	Inhibited apoptosis of NPC; Prevented M1 polarization and promoted M2 polarization.	[179]

**Table 3:** Specific miRNA-containing EV in vivo studies.

miRNA	Source of EVs	Application	Effect	Reference
miR-129-5p	BMMSCs	Rat tail IDD model	Increased Disc Height Index (DHI) and reduced apoptosis and ECM degradation.	[190]
miR-125-5p	CEPMSCs	Rat tail IDD model	Inhibited apoptosis of NPCs.	[191]
miR-125a-5p	Rat MSCs	Rat IDD model	Attenuated IDD.	[192]
miR-125b-5p	BMMSCs	Rat IDD model	Decreased Pfirrmann scores and prevented degradation of ECM.	[193]
miR-133a-3p	CEP chondrocytes	Rad IDD model	Decreased loss of disc height and improved MRI and histological scores.	[194]
miR-17-5p	BMMSCs	Rat IDD model	Alleviated IDD.	[195]
miR-217	BMMSCs	Rat IDD model	Increased DHI, reduced apoptosis of NPCs, and reduced ECM degradation.	[172]
miR-31	BMMSCs	Mice IDD model	Reduced inflammation and apoptosis.	[196]

**Table 4:** EVs and MSCs in clinical trials for stroke.

Trial ID	Therapy Type	Stroke Type
NCT01678534	MSC—allogeneic adipose-derived	Acute ischemic stroke
NCT01297413	MSC—allogeneic bone marrow-derived	Chronic ischemic stroke
NCT00875654 (ISIS-HERMES)	MSC—autologous bone marrow-derived	Subacute ischemic stroke
NCT01716481 (STARTING-2)	MSC—autologous bone marrow-derived, expanded with acute-phase serum	Ischemic stroke with persistent deficits
NCT01461720	MSC—autologous bone marrow-derived	Middle cerebral artery infarcts
NCT03371329	MSC—IV and intrathecal BM-MSCs	Intracerebral hemorrhage (ICH)
NCT05292625	MSC—cord-derived	Post-stroke sequelae
NCT04063215	MSC—autologous adipose-derived	Traumatic brain injury/Hypoxic–ischemic encephalopathy
NCT02564328	MSC—autologous bone marrow-derived	Chronic ischemic stroke
NCT04434768	MSC—umbilical cord-derived	Acute ischemic stroke
NCT05850208	MSC—autologous bone marrow-derived	Ischemic stroke
NCT04590118 (ASSIST)	MSC—allogeneic	Chronic stroke
NCT06862388	MSC—umbilical cord-derived	Subacute ICH
NCT06518902	MSC—cord-derived, repeated	Acute ischemic stroke
NCT04093336	MSC—cord-derived, single infusion	Acute ischemic stroke
NCT06997939	MSC—autologous bone marrow-derived	Ischemic stroke, motor recovery
NCT06129175 (Stroke Neuroncell-EX)	MSC—allogeneic cord-derived	Acute ischemic stroke
NCT02580019	MSC—cord-derived, repeated	Acute ischemic stroke
NCT03384433	EV—MSC-derived exosomes, miR-124	Acute ischemic stroke
NCT05370105	EV—circulating EVs (observational)	Stroke rehabilitation
NCT06319742 (ElViS-ACS)	EV—circulating EV profiling	Ischemic stroke, TIA, stroke mimics
NCT05645081	EV—endothelial-derived EV profiling	Ischemic stroke
NCT06871800 (PRISMA)	EV—blood EV profiling	Rehabilitation after stroke, severe brain injury
NCT06995625 (STEVIA)	EV—stem cell-derived EV therapy SNE-101	Acute ischemic stroke
NCT05158101	EV—intranasal umbilical cord MSC exosomes (AlloEx)	Stroke
NCT07143786	EV—induced neural stem cell-derived exosomes (iNSC-EV01)	Acute ischemic stroke
NCT06138210 (ExoCURE)	EV—hiPSC-derived exosomes (GD-iExo-003)	Acute ischemic stroke
NCT06612710	EV—induced neural stem cell-derived exosomes (NouvSoma001)	Acute ischemic stroke

**Table 5:** Role of extracellular vesicles in autoimmune diseases.

Disease	Extracellular Vesicle Contribution to Pathogenesis
Multiple Sclerosis (MS)	EVs cross the blood–brain barrier and participate in inflammatory cascades, promoting immune cell migration into the central nervous system.
Rheumatoid Arthritis (RA)	EVs contribute to inflammation and joint destruction by stimulating the formation of immune complexes and promoting cartilage degradation. EVs can also carry citrullinated proteins, a key marker of RA autoimmunity, and present them to T-cells, contributing to disease pathogenesis. Autophagy also contributes to the generation of autoantigens and EVs. Plasma exosomes from RA patients show elevated miRNAs, such as let-7a-5p and miR-25-3p, demonstrating high diagnostic accuracy.
Systemic Lupus Erythematosus (SLE)	EVs bearing self-antigens form immune complexes and trigger proinflammatory responses. Elevated EV levels correlate with disease activity, and urinary exosomal miRNA profiles can indicate kidney impairment and predict lupus nephritis progression, suggesting their role in disease pathogenesis and their utility as diagnostic markers.
Sjögren’s Syndrome (SS)	EVs contain autoantigens, such as Ro/SSA and La/SSB, reflecting salivary gland pathology and offering diagnostic potential.
Type 1 Diabetes (T1D)	EVs from pancreatic cells deliver autoantigens to autoreactive T- and B-cells, contributing to the autoimmune destruction of β-cells.
Autoimmune Thyroid Disease (AITD)	Patient-derived microvesicles suppress regulatory T-cell differentiation and induce inflammatory mediators, promoting disease progression, which highlights a key role in the disease’s pathogenesis.

**Table 6:** Extracellular vesicles as potential diagnostic markers in autoimmune diseases.

Disease	EVs as Potential Biomarkers
Primary Sjögren’s Syndrome (pSS)	Patients with pSS have increased levels of microparticles (MPs) and endothelial microparticles (EMPs) in their plasma. Specific proteins and miRNAs, such as adipocyte plasma membrane-associated protein, SIRPA, LSP1, Copine 1, and miRNAs like hsa-mir-768-3p, have also been found to be either upregulated or serve as promising biomarkers in the saliva and tears of pSS patients. Salivary EVs from patients with pSS show differentially expressed proteins, including members of the S100 protein family, Annexin A2, and CD14, which could be used for diagnosis and treatment monitoring.
Systemic Lupus Erythematosus (SLE)	Total MP levels are increased in the plasma of SLE patients, with specific types from platelets, monocytes, and T-cells also elevated. These may be associated with disease duration and cardiovascular disease risk. Additionally, certain types of IgG-harboring MPs correlate with disease activity.
Oral Lichen Planus (OLP)	EVs from the plasma of OLP patients can enhance T-cell proliferation and reduce apoptosis. The altered expression of miRNAs, including hcmv-miR-UL59, miR-4484, and miR-34a-5p, has been identified as a potential biomarker for OLP, with the level of miR-34a-5p correlating with disease severity.
Behçet’s Syndrome (BS)	Patients with BS have increased levels of total MPs, platelet-derived MPs, and procoagulant MPs, suggesting a role in hemostatic system activation.
Type 1 Diabetes Mellitus (T1DM)	EVs can transfer autoantigen peptides from insulin-producing cells, which is a key part of the disease’s pathogenesis. Upregulated miRNAs, such as miR-16-5p, miR-574-5p, and miR-302d-3p, are found in the plasma of T1DM patients. Additionally, insulin-containing EVs and their cargo, like islet autoantigens and glutamic acid decarboxylase 65 (GAD65), can indicate the destruction of pancreatic cells.
Rheumatoid Arthritis (RA)	The number of EVs is significantly higher in the plasma and synovial fluid of people with RA compared to healthy individuals. Elevated levels of certain miRNAs in EVs, such as miR-212-3p, miR-338-5p, miR-410-3p, and miR-537, have been found in the early stages of RA. The expression profiles of long non-coding RNAs (lncRNAs) in EVs from the synovial fluid of RA patients differ significantly from those with osteoarthritis (OA) and gout. Specific lncRNAs, including SNHG6, RPS18P9, and CXXC4-AS1, have been identified as potential biomarkers for RA diagnosis. EVs from RA patients have shown differentially expressed proteins, such as stromelysin-1 and ezrin, which correlate with disease activity. EVs can also carry citrullinated proteins, a key marker of RA autoimmunity, and present them to T-cells, contributing to disease pathogenesis. Autophagy also plays a role in generating these autoantigens and EVs.
Inflammatory Bowel Disease (IBD)	Specific proteins within EVs, such as Annexin A1, show elevated levels in the serum of patients with active IBD, correlating with the severity of mucosal inflammation. This suggests EVs can serve as a reliable, non-invasive tool to monitor disease progression. MicroRNAs encapsulated within EVs, such as miR-200b-3p, have been shown to be upregulated in models of acute colitis, indicating their potential as diagnostic markers for active disease. Salivary exosomal proteins like proteasome subunit alpha type 7 (PSMA7) and serum exosomal proteins like pregnancy zone protein (PZP) are found at significantly elevated levels, indicating their potential as biomarkers for IBD.

## Data Availability

No original data was generated in this article.
